# Skin microbiome-biophysical association: a first integrative approach to classifying Korean skin types and aging groups

**DOI:** 10.3389/fcimb.2025.1561590

**Published:** 2025-07-07

**Authors:** Seyoung Mun, HyungWoo Jo, Young Mok Heo, Chaeyun Baek, Hye-Been Kim, Haeun Lee, Kyeongeui Yun, Jinuk Jeong, Wooseok Lee, Dasom Jeon, So Min Kang, Seunghyun Kang, Young-Bong Choi, Sangjin Han, Gabriel Kim, Kung Ahn, Dong Hun Lee, Yong Ju Ahn, Dong-Geol Lee, Kyudong Han

**Affiliations:** ^1^ Department of Microbiology, College of Science & Technology, Dankook University, Cheonan, Republic of Korea; ^2^ Center for Bio-Medical Engineering Core Facility, Dankook University, Cheonan, Republic of Korea; ^3^ Department of Cosmedical Materials, College of Bio-convergence, Dankook University, Cheonan, Republic of Korea; ^4^ Research & Innovation (R&I) Center, COSMAX BTI, Seongnam, Republic of Korea; ^5^ HuNBiome Co., Ltd., Research and Development (R&D) Center, Seoul, Republic of Korea; ^6^ Department of Bioconvergence Engineering, Dankook University, Yongin, Republic of Korea; ^7^ Department of Clinical Research, Korea Biomedical Research Institute, Seongnam, Republic of Korea; ^8^ Center for Digital Health, Medical Science Research Institute, Kyung Hee University Medical Center, Kyung Hee University College of Medicine, Seoul, Republic of Korea; ^9^ Department of Dermatology, Seoul National University Hospital, Seoul National University College of Medicine, Seoul, Republic of Korea

**Keywords:** dermatologic conditions, microbiome, Korean skin cutotypes, skin microbiome-biophysical association, core genera

## Abstract

**Introduction:**

The field of human microbiome research is rapidly expanding beyond the gut and into the facial skin care industry. However, there is still no established criterion to define the objective relationship between the microbiome and clinical trials for developing personalized skin solutions that consider individual diversity.

**Objectives:**

In this study, we conducted an integrated analysis of skin measurements, clinical Baumann skin type indicator (BSTI) surveys, and the skin microbiome of 950 Korean subjects to examine the ideal skin microbiome-biophysical associations.

**Methods:**

By utilizing four skin biophysical parameters, we identified four distinct Korean Skin Cutotypes (KSCs) and categorized the subjects into three aging groups: the Young (under 34 years old), the Aging I group (35-50), and the Old group (over 51). To unravel the intricate connection between the skin’s microbiome and KSC types, we conducted DivCom clustering analysis.

**Results:**

This endeavor successfully classified 726 out of 740 female skin microbiomes into three subclusters: DC1-sub1, DC1-sub2, and DC2 with 15 core genera. To further amplify our findings, we harnessed the potent capabilities of the CatBoost boosting algorithm and achieved a reliable framework for predicting skin types based on microbial composition with an impressive average accuracy of 0.96 AUC value. Our study conclusively demonstrated that these 15 core genera could serve as objective indicators, differentiating the microbial composition among the aging groups.

**Conclusion:**

In conclusion, this study sheds light on the complex relationship between the skin microbiome and biophysical properties, and the findings provide a promising approach to advance the field of skincare, cosmetics, and broader microbial research.

## Introduction

Skin clinical research trials are conducted to study the effectiveness and safety of a new product or formulation to measure the condition of a subject’s skin. Rising consumer demand for personalized skincare drives clinical research into the skin microbiome to develop tailored solutions (Goyal et al., 2022; Gupta et al., 2022). Skin microbiome studies explore its role in skin health, addressing diverse phenotypes across age groups. These efforts aim to create effective, evidence-based products for preventing skin aging (Elder et al., 2021; Souak et al., 2021; Borja Guerrero et al., 2022; Dhandapani et al., 2023).

The Baumann Skin Type Indicator (BSTI) classifies skin phenotypes based on oiliness, sensitivity, pigmentation, and elasticity, serving as a key tool in dermatological research (Baumann, 2008). However, its reliance on self-assessment often leads to inaccuracies, particularly for Asians, where Korean studies show discrepancies in pigmentation and sensitivity (Ahn et al., 2017; Lee et al., 2019). Advanced clinical measurement devices, such as corneometers and cutometers, provide precise, non-invasive assessments of skin parameters like hydration, elasticity, and transepidermal water loss (Tkaczyk, 2017; Seo et al., 2022). Skin clinical measurement devices are tools used to evaluate and quantify various skin parameters, such as hydration, elasticity, and pigmentation (Seo et al., 2022). Integrating these objective metrics with refined BSTI questionnaires improves the accuracy of skin phenotype determination for diverse populations.

Along with the growth and interest in dermatology, the microbiome, which includes all the microorganisms that inhabit the human body, has become an increasingly popular research topic in recent years. The gut microbiome, in particular, has received a lot of attention due to its potential influence on overall health and disease. However, skin microbiomes are also an important area of research, as they play a vital role in skin health and function. Age-related changes in the skin microbiome are highly variable and can be influenced by both personal and external environmental factors. Therefore, the skin microbiome, increasingly vital in cosmetics and dermatology, is shaped by age, sex, genetics, and environment, driving personalized healthcare innovations (Byrd et al., 2018). Its diverse microbial communities protect against pathogens and maintain skin barrier function, critical for skin health. Disruptions in this microbiome are linked to disorders like acne, eczema, and psoriasis, spurring research and commercialization (Dréno et al., 2016).

Increased *Cutibacterium acnes* (*C. acnes*) abundance is linked to acne-prone skin, prompting targeted skincare interventions (Rozas et al., 2021). Atopic dermatitis patients exhibit reduced skin microbiome diversity and elevated *Staphylococcus aureus* (*S. aureus*), exacerbating inflammation (Kong et al., 2012). Chronic wound patients have less diverse microbiomes with more pathogenic bacteria, while *Staphylococcus epidermidis* (*S*. *epidermidis*) promotes wound healing (Wolcott et al., 2016). In diabetes, a less diverse microbiome with increased *Staphylococcus* species increases the risk of skin infection (Gardiner et al., 2017).

The clinical relevance of the skin microbiome has been well documented, with many studies suggesting that the microbiome plays an important role in skin health and disease, but there is a lack of research on the association between skin type and the skin microbiome in normal human subjects, limited by environmental and ethnic variables. Only a few studies have shown an association with different skin types, which are categorized by factors such as oiliness, dryness, and sensitivity. Oily skin has been found to have a different skin microbiome compared to dry skin. Specifically, there is a higher abundance of *Propionibacterium acnes* (*P. acnes*) on oily skin. Dry skin has been associated with a less diverse skin microbiome and a decrease in certain beneficial bacteria, such as *Staphylococcus epidermidis* (Mukherjee et al., 2016). Additionally, dry skin may be more susceptible to colonization by pathogenic bacteria. Sensitive skin has a distinct microbiome with a higher abundance of certain bacteria, exacerbating skin inflammation (Tay et al., 2021). Combination skin has a more diverse microbiome, but microbial communities can still vary across different areas of the skin. Systematic clinical and molecular genetic studies must be completed to better understand the specific microbiome associated with different skin types and to develop targeted interventions to improve skin health.

Thus, this study aimed to address dermatological concerns by building a comprehensive meta-database of Korean facial skin, questionnaires (including Baumann), incorporating clinical measures, and skin microbiome data from 950 participants of both genders (756 female and 194 male) and all ages (0-88). Two aging points with contrasting upper and lower group proportions in pigmentation, elasticity, and oiliness values are demonstrated to differentiate three aging groups: Young, Aging I, and Old, based on six skin measurements. Accordingly, we defined four skin types based on clinically measured metrics, such as hydration, oiliness, pigmentation, elasticity, pores, and wrinkles, which could distinguish skin phenotypes in each group. Using non-invasive techniques, we analyzed the relationship between microorganisms and clinical metrics, defining 15 core and representative genera for each of the 12 skin phenotypes via bioinformatic analysis and artificial intelligence (AI) machine-learning experiments. Additionally, we identified the significant microorganisms with potential skincare applications through pathway enrichment analysis. These findings provide a foundation for future skin microbiome studies to discover microbial materials and functions relevant to skin health. Furthermore, our study’s results may be valuable in developing an effective and tailored cosmetic solutions using the skin microbiome database.

## Materials and methods

### Sample collection

The facial rinse sample collection in this study (skin microbiota samples collection) was approved by the ethics committee of H&BIO Corporation R&D CENTER (H&Bio, South Korea) Institutional Review Board (IRB Protocol Number: HBABN01-210217-HR-0181-01). All clinical processing applied in this study was conducted under the guidelines and regulations of the Helsinki Declaration. A total of 950 samples were collected from the entire faces of healthy Koreans (756 females and 194 males) without chronic skin diseases, such as atopic dermatitis and psoriasis. These subjects are Koreans living in the same area, aged 0 to 88. They were asked not to wash or use any formulation on their face for 12 h to 18 h before sampling. Applicants have stayed at 22 ± 2°C, 50 ± 5% of humidity for 20 min to normalize the skin condition. The facial rinse samples for subjects were collected using 500 mL of sterilized distilled water for 2 min, and the collected samples were immediately frozen at -20°C prior to DNA extraction or proceeded with microbial genomic DNA extraction. All subjects completed a 60-item dermatological clinical self-assessment questionnaire (**Supplementary Table 1**).

### Microbial genomic DNA extraction and

A 150 mL aliquot of the rinse solution was filtered through a Corning^®^ 150 mL Vacuum Filter/Storage Bottle System with a 0.22 µm polyethersulfone (PES) membrane (Corning, Cat. No. 431097) using a vacuum pump after mixing gently. Microbial genomic DNA was extracted from the 0.22 µm PES filter membranes using the QIAamp PowerFecal Pro DNA Kit (Qiagen, Germany), following the manufacturer’s instructions with modifications for filter-based samples. The quality of all extracted bacterial gDNA was assessed using the Qubit 4 (ThermoFisher Scientific, USA). The extracted mDNA samples were then stored at 4°C until further processing.

### Determination of Baumann skin type indicator

The Baumann Skin Type Indicator (BSTI) is a self-administered questionnaire that classifies skin into 16 types based on four categories: dryness/oiliness, sensitivity/resistance, pigmentation/non-pigmentation, and wrinkles/elasticity (Baumann, 2008; Ahn et al., 2017; Lee et al., 2019). Participants answer 60 questions, scoring 1–4 points per question (2.5 points for unanswered questions). Scores are tallied separately for each category to determine skin type. For dryness/oiliness (11 questions), scores ≥27 indicate oily skin, and ≤26 indicate dry skin. For sensitivity/resistance (18 questions), scores ≥30 (plus 4 points for specific skin conditions) indicate sensitive skin, and ≤29 indicate resistant skin. For pigmentation/non-pigmentation (10 questions), scores ≥31 (plus 5 points if sunlight aggravates moles) indicate pigmented skin, and ≤30 indicate non-pigmented skin. For wrinkles/elasticity (20 questions), scores ≥41 (plus 5 points if aged >65) indicate wrinkly skin, and ≤40 indicate elastic skin. Combining scores from all categories identifies the participant’s BSTI skin type. For pediatric and adolescent subjects (aged 0–18 years), skin characteristic assessments, including Baumann Skin Type Indicator (BSTI) evaluations, were derived from clinical examinations by dermatologists at the Center for Digital Health, Kyung Hee University Medical Center, supplemented by proxy questionnaires completed by the subjects’ parents.

### Biophysical measurement for skin conditions

To characterize the facial skin properties of participants, a series of non-invasive measurements were conducted at baseline to assess epidermal hydration, transepidermal water loss (TEWL), sebum production, wrinkles, pores, skin color/tone, elasticity, and dermal density. But, Wrinkle measurements (lateral canthal lines and nasolabial folds) were not performed for pediatric subjects (aged 0–9 years) due to the absence of detectable wrinkles and technical difficulties. All measurements were performed under controlled conditions (temperature: 22 ± 2°C, relative humidity: 50 ± 5%) following a 12-hour period without face washing or cosmetic application to ensure consistency. Measurements were conducted in triplicate (unless specified otherwise), and average values were analyzed to account for variability. Detailed protocols for dermatological assessments are described in **Supplementary Materials 1**.

### Integrating clinical skin measurements

In order to ensure accurate skin quantification in localized areas, several measures were taken. Moisture content and water loss were measured in triplicate, and the average values were used in the analysis to minimize errors. Separate measurements were taken for the forehead and cheeks to determine water content and water loss. Their averages were used to represent an individual’s overall water content and Transepidermal Water Loss (TEWL). Pores were also measured on both cheeks, and their average value was used to represent the severity of an individual’s pores. The other measurements for the amount of skin sebum, wrinkles, tone, elasticity, and dermal density were performed by individual devices. Using these measures can provide a more accurate representation of an individual’s skin condition, which is crucial in developing effective skincare routines and treatments. A detailed calculation method and calculated formulas are described in Supplementary Information (**Supplementary Material 2**).

### A skin biophysical multiparameter-based prediction model for the aging group classification

To construct and evaluate our classification models, we used the PyCaret library (version 2.3.6) in Python (version 3.8.12) (Ali, 2020). Prior to model construction, our samples were split into an 80% training set and a 20% test set, with ‘data_split_stratify=True’ (and ‘data_split_stratify=[target_variable]’) to ensure that the ratio of target variables was maintained in both sets. Predictive models were constructed using the CatBoost algorithm, which is a boosting algorithm that has shown high performance in many recent classification problems (Prokhorenkova et al., 2018). The hyperparameters of our models were tuned using a random grid search algorithm, based on the scikit-learn library (version 0.23.2), through five-fold cross-validation (Pedregosa et al., 2011). To account for imbalanced sample numbers for the target variable, we evaluated the performance of our models through three types of Receiver Operating Characteristic (ROC) curve: (1) an ROC curve for each class, (2) a micro-average ROC curve for all classes, and (3) a macro-average ROC curve for all classes (Pedregosa et al., 2011). The micro-average method reflects each prediction equally, while the macro-average method treats all classes equally when calculating the average.

### Amplicon library construction for 16S V3-V4 illumina sequencing

To prepare the 16S amplicon libraries for Next Generation Sequencing (NGS) based metagenome sequencing, the official Illumina 16S metagenome library construction protocol was followed, targeting the V3-V4 hypervariable region of the bacterial 16S rRNA gene (Illumina, USA) (Bukin et al., 2022). PCR amplification for sequencing library construction was performed using the 16S V3-V4 hypervariable region-specific universal primer pair and 2X KAPA HiFi Hot Start Ready Mix (Roche, Germany). The forward and reverse primer sequences were 16S 341F (5’-TCGTCGGCAGCGTCAGATGTGTATAAGAGACAGCCTACGGGNGGCWGCAG-3’) and 16S 806R (5’-GTCTCGTGGGCTCGGAGATGTGTATAAGAGACAGGACTACHVGGGTATCTAATCC-3’), respectively. After PCR amplification, all amplicon products were purified using AMPure XP beads (Beckman Coulter, USA). An additional PCR amplification was performed to add the Illumina adapter and multiplex indices using the Nextera XD Index (Illumina, USA). The final PCR products were purified once again using the AMPure XP beads. Following library preparation, the 16S V3-V4 metagenome sequencing was carried out using the Illumina Miseq (2 x 300) paired-end sequencing workflow (Illumina, USA).

### Data analysis of skin microbiome

The skin microbiome sequence data obtained through MiSeq was analyzed using the QIIME™ 2 pipeline plugin (2020.11). To improve the accuracy of classification and annotation results, Figaro (https://github.com/Zymo-Research/figaro) was used to remove low-quality reads and adapters before implementing DADA2 (Divisive Amplicon Denoising Algorithm 2) in QIIME2 (Callahan et al., 2016; Bolyen et al., 2019). This can be achieved by setting a specific quality score, sliding a window of quality scores, or removing reads shorter than a certain length. DADA2 is a powerful bioinformatics tool that uses a denoising algorithm to correct sequencing errors and cluster reads into Amplicon Sequence Variants (ASVs) based on their sequences. The ASVs were then subjected to bacterial classification using a Naïve Bayes classifier based on V3-V4 hypervariable reads extracted from the SILVA 138v 99% rRNA database to improve accuracy. ASVs annotated with Archaea, Eukaryotes, mitochondria, or chloroplasts were removed to focus only on bacterial taxa. The ASVs were aligned using the phylogeny align-to-tree-mafft-fasttree plugin and subjected to α-diversity (observed features, Chao1 index, Shannon’s index, Simpson’s index, Pielou’s evenness) and β-diversity (Bray-Curtis, Unweighted unifrac, generalized UniFrac) analysis, which produced 1,391 rarefied depth reads. To further analyze the data, the samples were divided into an appropriate number of clusters using the ASVs abundance table and a generalized UniFrac distance matrix with the DivCom (Divide and Compare) tool (Intze and Lagkouvardos, 2022). Finally, the metagenomic functional composition of the taxa abundance was inferred using PICRUSt2 from ASVs sequence and table (Douglas et al., 2020). Overall, the combination of these methods and tools allowed for a comprehensive analysis of the skin microbiome data, including accurate classification, diversity analysis, clustering, and functional inference.

### Statistical analysis & machine learning experiments

Correlation between clinical metrics and between skin microbes was confirmed by Spearman’s rank correlation coefficient in R, and visualization was performed using the corrplot package (https://github.com/taiyun/corrplot). Group comparison of α-diversity and taxonomies was evaluated for statistical significance using Kruskal-Wallis and Mann-Whitney (Wilcoxon-test rank sum test). To confirm the similarity between groups in distance matrices from β-diversity metrics, Principal Coordinate Analysis (PCoA) was performed through the QIIME2 plugin (Bolyen et al., 2019). Permutational analysis of variance (PERMANOVA) analysis was also conducted using the pairwise Adonis package in R, with 999 permutations (Martinez Arbizu, 2020). Linear Discriminant Analysis Effect Size (LEfSe) was calculated with LDA score ≥ 2.0 to identify significant taxonomy within each group (Chang et al., 2022). Additionally, Analysis of Compositions of Microbiomes with Bias Correction 2 (ANCOM-BC2) was utilized to determine if a specific genus was differentially abundant between KSCs (Lin and Peddada, 2020). For 15 selected genera, heatmap plots of negative log10-transformed p-values from all possible pairwise comparisons were generated. To distinguish each type from the other three types of the same aging group, a prediction model was constructed for every single type using CatBoost, with 15 selected genera as features (Prokhorenkova et al., 2018). The samples were divided into an 80% training set to build the model and a 20% test set to validate the model. Synthetic Minority Oversampling TEchnique (SMOTE) was applied to the training set to overcome class-imbalance bias (Chawla et al., 2002). The hyperparameters were optimized using the Optuna framework (Akiba et al., 2019). Feature importance values were used to identify the genera associated with the types. The all detailed codes are opened at https://github.com/HuNBiome/KSC-Microbiome-2023.

### Availability of data and materials

The data supporting these findings have now been uploaded to the European Genome-phenome Archive (EGA: https://ega-archive.org/) for sharing by a number of researchers (EGA number: EGAS00001007334). Code for the pairwise comparison tests of 12 groups (3 age groups x 4 KSC types) for 15 genera and building ML models to classify groups using 15 genera as features are available at https://github.com/HuNBiome/KSC-Microbiome-2023. It will be freely downloaded.

## Results

### Recruitment and BSTI administration for skin typing

**Figure 1:**
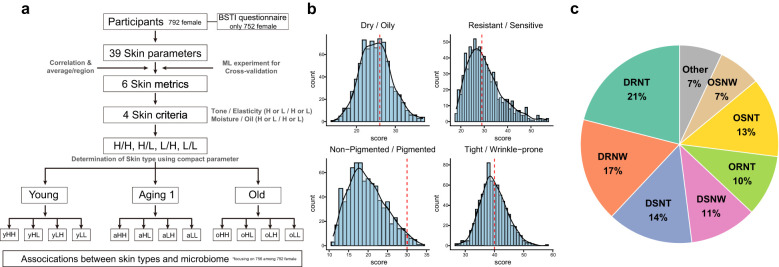
Screening Korean Skin Cutotypes (KSC): Research strategies and BSTI analysis unveiled. **(a)** This diagram represents the sequence of steps in determining KSC type for a total of 756 female subjects. Based on 39 skin clinical measurement data parameters, four skin criteria were finally selected and four KSC types were determined. **(b)** Distribution of female subjects based on sebum production, skin sensitivity, pigmentation, and wrinkle severity categories. The x- and y-axis represent the calculated score in each category and the accumulated number of subjects, respectively. The red dashed line is the median. **(c)** Determined proportions of 16 Bauman types in 752 female subjects. Detailed BSTI results for each subject are presented in **Supplementary Table 1**.

Our study enrolled 989 Koreans (792 females, 197 males) for comprehensive assessments, including skin measurements, female-only questionnaires on lifestyle and cosmetic use, and microbiome sampling via face wash. The resulting Korean facial skin database comprised BSTI survey data, skin measurements, and microbiome profiles (**Table 1**; **Supplementary Table S1**). Using clinical skin type classifications, we analyzed microbiota distribution across types. This prospective study investigated skin condition-microbiome relationships in 756 female participants with a robust approach (**Figure 1a**).

**Table 1:** Number of subjects by gender and age.

**Female**	**00s**	15	**Male**	**00s**	14
	**10s**	82		**10s**	21
	**20s**	110		**20s**	27
	**30s**	105		**30s**	22
	**40s**	106		**40s**	21
	**50s**	103		**50s**	19
	**60s**	104		**60s**	26
	**70s**	105		**70s**	24
	**80s**	62(26)		**80s**	23

Using the Baumann Skin Type Indicator (BSTI), we classified the skin types of 752 Korean female participants into 16 categories based on four dichotomous traits: dryness/oiliness, sensitivity/resistance, pigmentation/non-pigmentation, and wrinkles/tightness (**Supplementary Table S1**). The distribution showed 64% with dry skin, 36% with oily skin; 53.5% with resistant skin, 46.7% with sensitive skin; 97.7% with non-pigmented skin, 2.3% with pigmented skin; and 58.4% with tight skin, 41.6% with wrinkled skin (**Figure 1b**). The most common skin type was DRNT (20.6%, n=155), followed by DRNW (17.3%, n=130), DSNT (14.1%, n=106), OSNT (13.3%, n=100), DSNW (10.8%, n=81), ORNT (9.7%, n=73), OSNW (7.4%, n=56), ORNW (4.7%, n=35), DRPW (0.7%, n=5), OSPW (0.5%, n=4), and DSPT (0.3%, n=2). DRPT, DSPW, ORPT, ORPW, and OSPT each had one participant (0.1%) (**Figure 1c**).

### Selection of representative biophysical metrics for skin condition assessment

It is possible that the choice of factors or variables used in a skin BSTI is arbitrary. It is also possible that the results will vary depending on which of the various factors that assess skin health are used. For this reason, a ganzheitliche (holistic) approach to assessing and improving skin health is required, taking into account a variety of factors. To robustly evaluate skin health, we conducted a comprehensive assessment of 989 Koreans (792 females, 197 males), measuring 39 biophysical parameters across nine categories: oiliness, hydration, water loss, skin tone, elasticity, density, nasolabial folds, lateral canthal lines, and pores.

**Figure 2:**
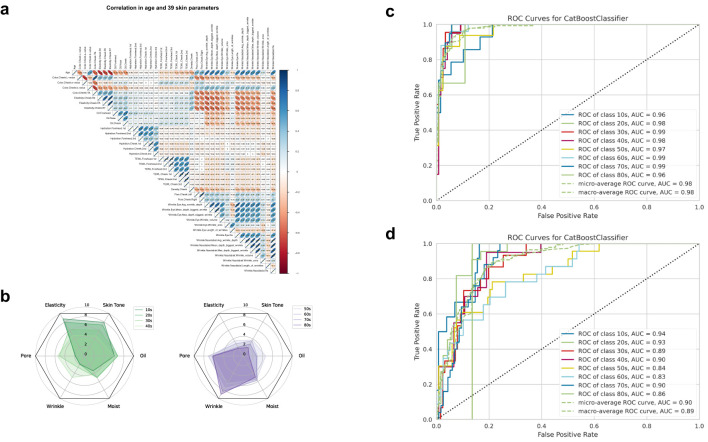
Assessment of key metrics selection and predictive performance using biophysical parameters. **(a)** Correlation analysis through the Pearson coefficient method between each of the 39 biophysics parameters. As shown in the scale bar on the right, blue and red colors represent positive and negative correlations, respectively, with higher correlations being darker. **(b)** Polygonal chart of age-related changes in six metrics representing skin condition (elasticity, skin tone, oil, pore, wrinkle, moisture). **(c)** Mean receiver operating characteristic (ROC) curves for age estimation using 39 biophysical parameters and figure **(d)** the representative six metrics. ROC curves were used to compare the predictive power of the six metrics. The Area Under the ROC Curve (AUC) scores for each age group are noted in the legend and represented by different colors. The green dashed line indicates the expected AUC for a random chance classifier. Plots depict the tradeoff between true-positive and false-positive. The closer the curve follows the left-hand border and the top border of the ROC space, the more accurate the test.

Three principles guided parameter selection: (1) averaging repeated, site-specific measurements to enhance reliability; (2) combining hydration and water loss into a single moisture metric; and (3) selecting representative parameters based on statistical correlations. This holistic approach ensures valid, reliable metrics for skin-microbiome correlations (**Figure 2a**; **Supplementary Table 2**).

For skin tone, the ITA (Individual Typology Angle) metric, which combines lightness (L*), greenness (a*), and yellowness (b*), was selected as a representative metric. Among the skin elasticity parameters, R7 showed a high correlation (Pearson Correlation Coefficient, PCC) with other elasticity parameters, R2 and R5 (R7-R5: 0.91, R7-R2: 0.92, R2-R5: 0.82). Thus, R7 was selected as a quantitative parameter representing elasticity since it is widely used in existing studies. Skin density (R: skin fold thickness) was excluded as a key parameter since R7 sufficiently represented it and showed moderate correlation (PCC) with other elasticity parameters (R7: 0.44, R: 0.36, R2: 0.37) (Ryu et al., 2008; Ohshima et al., 2013). The roughness of the skin surface (Ra value) was selected as a wrinkle-related parameter, precisely one nasolabial fold Ra. Additionally, since there was a high correlation between nasolabial folds and lateral canthal lines (0.61), we selected only one nasolabial fold Ra for the representative parameter (Yoo et al., 2016). Through a process of correlation and normalization, six indicators were selected as representative of an individual’s skin condition: oiliness, hydration, skin tone (ITA), elasticity (R7), average pore size (Pore), and nasolabial folds (Ra). This comprehensive set of metrics provides a more accurate and complete understanding of an individual’s skin condition.

### Validation of the selected metrics through machine learning-based age prediction

We employed a systematic evaluation process to determine the extent to which the six selected metrics accurately reflected the participants’ skin condition and how it changed with age. Our first step involved creating a polygonal chart that visually displayed the trends of each metric against age (**Figure 2b**). Our analysis of this chart revealed that skin elasticity and tone values were relatively higher in all age groups, with an average score of above 5, which is considered a relative average. Similarly, oiliness was also found to be above five from the teenage years to the 40s. However, we found that pores and wrinkles had an average value of more than 5 in the 40+ age group. In contrast, moisture levels exhibited a mean value ranging between 4 and 6 for all age groups, except for the teenage group, which had an average score of 3.48. Next, we compared the performance of a machine learning model that uses a full set of 39 parameters to predict age with a model that uses only six metrics to predict age. The Catboost algorithm, which has shown strong performance in recent AI competitions, was implicated in this evaluation (Zhang et al., 2020). When all 39 skin parameters were used to predict age, each age group had a ROC-AUC (The Receiver Operator Characteristic-Area Under Curve) score between 0.94 and 1, with a micro-average ROC-AUC score of 0.98 and a macro-average ROC-AUC score of 0.97. (**Figure 2c**) The performance of the model in predicting each participant’s age using only the six metrics resulted in ROC-AUC scores between 0.85 and 0.94 for each age group, with a micro-average ROC-AUC score of 0.91 and a macro-average ROC-AUC score of 0.90 (**Figure 2d**).

### Deriving four representative skin types using biophysical criteria

**Figure 3:**
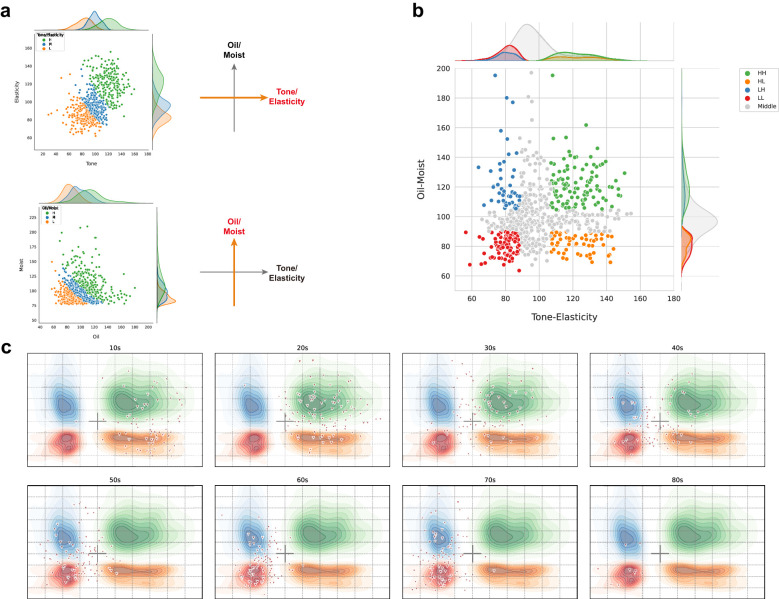
Determination and compartmentalization of the skin type for 705 Korean female. **(a)** Scatter plots of tone/elasticity (top) and oil/moisture (bottom) for the tertile of groups by each criterion. Each individual in the high, middle, and low values of the x- and y-axis criterion was plotted in green, blue, and orange, respectively. **(b)** Principal component analysis based on tone/elasticity and oil/moisture criterion values. The integrated coordinates of the skin criteria show a characteristic distribution across the four types based on the skin type determination strategy. Individuals in the middle range were marked in gray. For example, high values for both tone/elasticity and oil/moisture were annotated as HH and low values as LL. The mountain diagram indicates the distribution of number of the enriched individuals. **(c)** Age-dependent clockwise change in skin type. The green, orange, blue, and red contours represent the types HH, HL, LH, and LL, respectively. The triangles represent the number of Korean female subjects in each age group, demonstrating that the skin type determination criteria in this study are efficiently representative of skin clinical variation.

In order to classify skin types, we re-selected the criteria of oil, moisture, tone, elasticity, pore, and wrinkle based on six representative metrics (oiliness, hydration, skin tone (ITA), elasticity (R7), average pore size (Pore), and nasolabial folds (Ra)) from 39 biophysical parameters. To determine the best representative criteria among the three metrics (elasticity, pores, and wrinkles), we evaluated three metrics using Pearson correlation coefficient (PCC) analysis, identifying elasticity as the primary indicator for both pores and wrinkles (elasticity-pores: -0.54, elasticity-wrinkles: -0.50, pores-wrinkles: 0.31). Due to strong correlations, we merged skin tone with elasticity into a “tone/elasticity” criterion and oiliness with hydration into an “oil/moisture” criterion. When the entire sample was divided into tertiles for tone/elasticity and oil/moisture, there were clear fractionation differences (**Figure 3a**). Among the five complexes based on the two criteria (tone/elasticity and oil/moisture), we focused on four types, HH, HL, LH, and LL without the gray zone, which were not in the medium (M) range in either tone/elasticity or oil/moisture (**Figure 3b**). We defined each of the four groups as HH (high in both tone/elasticity and oil/moisture), HL (high in color/elasticity but low in oil/water), LL (low in both), and LH (low in color/elasticity but high in oil/water). These four types formed the basis for subsequent stratified analyses (**Figure 3b**). The analysis of biological age-related changes in skin parameters revealed a noticeable clockwise shift in the distribution of ages in the integrated coordinates of the skin indicators.

### Age-related shifts in skin characteristics and stratification of aging groups

Analysis of skin parameters across age groups showed younger participants (10s–20s) predominantly in the HH zone (high tone/elasticity, high oil/moisture), while older participants (60s–80s) were in the LL zone (low tone/elasticity, low oil/moisture) (**Figure 3c**). This trend is further supported by the skin condition of participants across age groups, particularly in terms of skin tone, which has been shown to generally darken with age (Yang et al., 2022). The median skin tone values of participants by age group were found to be 46.2 in 10s, 43.5 in the 20s, 40.6 in the 30s, 37.5 in the 40s, 35.8 in the 50s, 35.1 in the 60s, 33.1 in the 70s, and 32.2 in the 80s (**Supplementary Figure 1a**). Similarly, a gradual decline in skin elasticity was observed across age groups. The median elasticity values for each age group were as follows: 65.0 in 10s, 60.2 in the 20s, 51.5 in the 30s, 47.3 in the 40s, 43.8 in the 50s, 41.2 in the 60s, 39.0 in the 70s, and 38.0 in the 80s (**Supplementary Figure 1b**). Oil levels peaked in the 20s–30s (median: 37.3 in 30s) then decreased (5.7 in 80s) (**Supplementary Figure 1c**). While, moisture levels showed no clear age-related trend (median: 2.6–3.6 across ages) (**Supplementary Figure 1d**).

**Figure 4:**
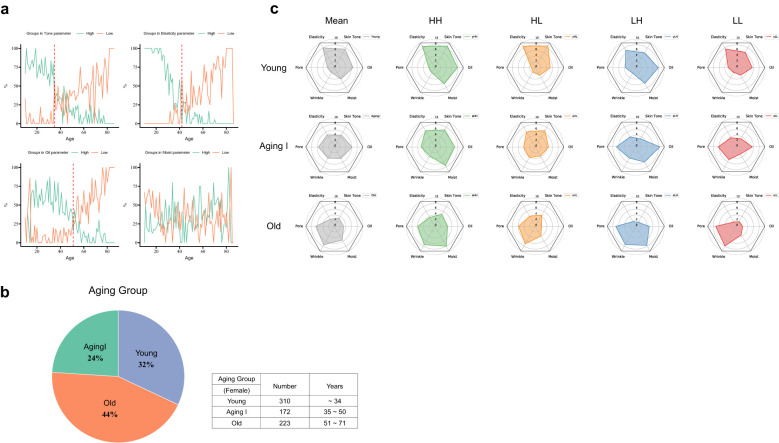
Turning points in skin condition and changes in skin type in aging groups. **(a)** The line chart represents the distribution of high and low skin type by age for 4 skin type criteria. The green and orange lines denote the proportion of subjects with high (positive) and low (negative) values in the age group. The red dotted lines indicate inflection points where tone, elasticity, and oiliness change. **(b)** Determination and the ratio of three Aging groups in skin turning points, based on four skin criteria. **(c)** Polygon chart for four skin types and biophysical variations by the Aging group. The green, orange, blue, and red polygons denote the types HH, HL, LH, and LL, respectively. The grey one is for the mean of each the Aging group.

In order to conduct an integrated analysis of all subjects based on the type classification of HH/HL/LH/LL, we rearranged the groups in the gray zone (MH, MM, ML, HM, LM) to be classified into the above four types (**Figure 3b**). In other words, participants with above-average values of the Tone-Elasticity (X-axis) and Oil-Moisture (Y-axis) value were classified as H, and those with below-average values were classified as L. As skin aging varies widely by individual and age, we grouped participants by age to investigate changes in these criteria. The results revealed distinct shifts at ages 35 and 51, marking key transitions in skin characteristics (**Figure 4a**). This age-based grouping clarified how skin types evolve. We found a reversal of the upper and lower groups in the Tone criteria at age 35. The proportion of the upper skin tone group decreased from 80% at age 34 to 27.3% at age 35, while the lower skin tone group surpassed the upper group, accounting for 54.5% at age 35. For the Elasticity criteria, the H and L groups became equal for the first time at age 36, both reaching 27.3%. In terms of the Oil criteria, there were two points of intersection between the upper and lower proportions at 14 and 51 years of age. We considered 51 years of age significant due to the trend of increasing oil volume until the age of 30 and then decreasing. At age 51, the proportion of the upper group was 14.3% and the proportion of the lower group was 28.6%. As for the Moisture criteria, we observed fluctuating proportions of the upper and lower groups from time to time. To minimize age bias error and age stratification error, we organized the aging groups based on the inflection points of the four skin criteria (**Figure 4a**). We categorized 310 subjects aged 34 or younger into the Young group, 172 subjects aged 35 to 50 into the Aging I group, and 223 subjects aged 51 or older into the Old group. These groups represented 31.6%, 22.4%, and 44% of all women, respectively, as shown in **Figure 4b**. We applied our skin type classification to each of these aging stages for further analysis.

### Skin type classification with the aging groups

**Figure 5:**
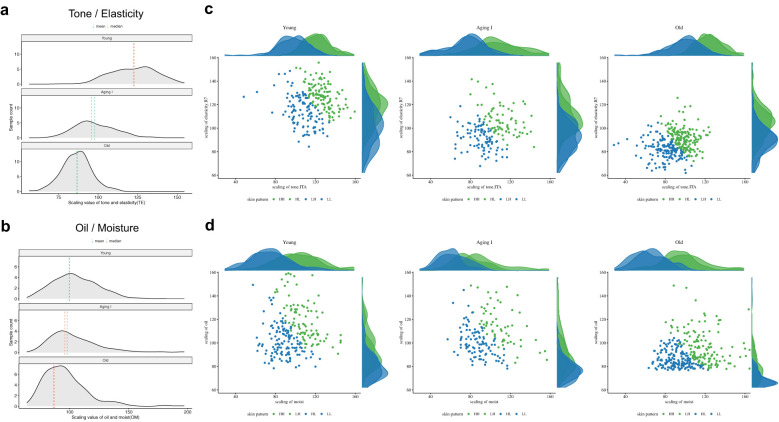
The skin-aging trends for four skin criteria dimensions. **(a)** Aging associations of tone and elasticity and figure **(b)** personal skin condition variations with oil and moisture. The binned graph shows the distribution of tone/elasticity or oil/moisture values and the number of samples per aging group. The green and red dashed lines indicate the mean and median values, respectively. This figure describes that among the key skin criteria, Tone/elasticity shows a sharp difference as getting old compared to oil/moisture. **(c, d)** Scatter plots representing relationships between tone/elasticity and between oil/moisture. In the three determined aging groups, tone/elasticity is converging to sharply lower values, while oil/moisture indicates an overall decrease in the oil criteria but similar values for the moisture criteria. Based on skin type with tone/elasticity and oil/moisture, the high group is colored green, and the low group is colored blue. The mountain diagram indicates the distribution of number of the enriched individuals.

We divided the Aging group (Young, Aging I, and Old) into the four skin types (HH/HL/LH/LL) and examined the differences between a total of 12 types in a polygonal chart (**Figure 4c**). We found significant differences in the mean values of the 6 metrics: skin tone, elasticity, pores, wrinkles, moisture, and oiliness for different skin types. Specifically, the yHH and yHL types had mean values above 8.7 for skin tone and elasticity, while the oLH and oLL types had mean values between 1.7 and 1.9. The mean values for the other eight groups were distributed between 3.3 and 7.6. As for oiliness, the aLH type had the highest mean value of 8.4, while the oHL and oLL types had the lowest mean value of 2.2. As for moisture, the mean values for the young group were yHH/yLH/yHL/yLL, and the same order was maintained in the Aging I and Old groups: aHH/aLH/aHL/aLL, oHH/oLH/oHL/oLL. As for wrinkles, the oLL type had the highest mean value of 7.9, but the oHH type had a mean value of 7.4. Finally, as for pores, the oLH and oLL types showed a similar trend to skin tone and elasticity, with a mean value of over 7, while the yHH and HL types had a mean value of under 2.1. Similar to the age-specific results, we also observed a clear trend of decreased skin tone and elasticity, and increased pores and wrinkles, as the aging groups progressed. The oiliness criterion showed a similar mean value for the Young and Aging I groups, at 6.5 and 6.2, respectively. However, the Old group exhibited a significant difference with a mean value of 3.2, indicating a noticeable oiliness decrease with age. The moisture criterion showed a mean relative value of 5 between the aging groups, ranging from 4.6 to 5.5. This suggests that there is not a significant difference in moisture levels across different age groups. Overall, the yLL, aLH, and oHH types were closest to the mean values of the respective aging groups, suggesting that these Korean skin cutotypes (KSCs) are most representative of the skin conditions in each aging group (**Figure 4c**). We observed changes of criteria in tone/elasticity and oil/moisture according to aging groups. As expected, tone and elasticity gradually decreased from the Young group to the Old group, acting as an important factor in distinguishing age groups. Additionally, tone and elasticity move together in aging groups and KSC types. The H- type is more reflected in the elasticity criteria, while the L- type is more influenced by the tone. In contrast, the difference in oil and moisture according to aging groups was not dramatic, but a minor difference in oiliness was observed. The oil values of each age group show a downward trend. Those means that oil/moisture plays an important role in distinguishing individual phenotypic differences and KSCs across the aging groups (**Figure 5**).

### Dynamics of the Korean skin microbiome by gender and age

In 950 Koreans (756 females, 194 males), skin microbiome analysis revealed higher alpha-diversity in females, particularly those aged 50 or older. In females, the alpha-diversity gradually decreased up to their 40s and then increased in their 50s (p<0.01 for all indexes). In contrast, males (n=194) showed a decreasing trend in diversity from their 30s to their 50s. The difference between males and females was significant in their 20s and 30s (p<0.05) (**Supplementary Figures 2a, b**) In terms of microbial composition, there were significant differences (p < 0.001) in *Cutibacterium*, *Streptococcus*, *Staphylococcus*, *Rothia*, and *Neisseria* genera between females and males (**Supplementary Figure 2c**; **Supplementary Table 4**). Significant diversity differences were observed in the top 10 genus compositions of females and males according to age groups (**Supplementary Figure 2d**). These findings highlight dynamic age- and sex-driven microbial shifts, validated by beta-diversity analysis with PERMANOVA test (**Supplementary Figure S3**).

In 950 Koreans, skin microbiome analysis revealed significant sex differences in Streptococcus, Cutibacterium, Staphylococcus, Rothia, and Neisseria (**Supplementary Table S4**). In females, Rothia and Neisseria increased with age, while Cutibacterium decreased; in males, Parvimonas rose and Lactobacillales declined (**Supplementary Figures S4a, b**). From the age of around 30, five microbial genera showed a significant difference in the skin microbiota between females and males, with varying levels of increase and decrease. Moreover, the time point of changes in the abundance of specific microorganisms was strongly correlated with the inflection point of clinical changes in the skin above (**Figure 4a**). Notably, Staphylococcus decreased in females but increased in males with age (**Supplementary Figure S4c**). These dynamic shifts highlight age- and sex-driven microbiome variations. The detailed statistical summary of the skin microbiome is annotated in **Supplementary Table 4**.

### Microbial shifts across korean female aging group

**Figure 6:**
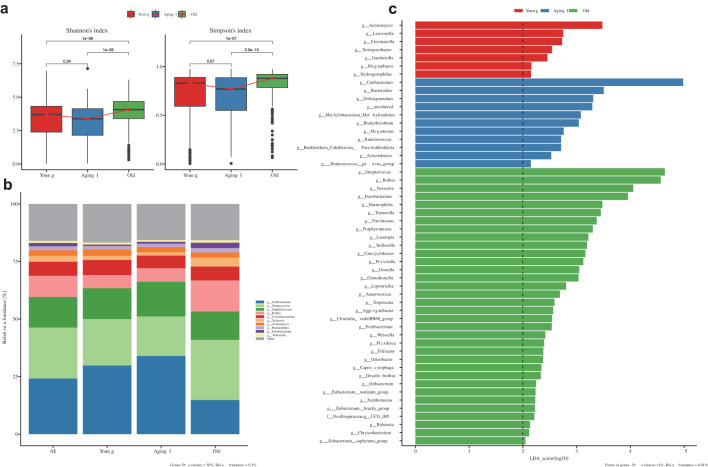
Comprehensive microbiome comparative analysis according to the decided Aging groups. **(a)** Box plot showing microbial alpha-diversity comparisons between three different age groups (Young, Aging 1, and Old groups). Shannon and Simpson alpha-diversity indices were applied to this alpha-diversity estimation, and it is a measure for confirming the microbial richness and evenness in each group (‘p’ means the p-value). **(b)** Relative abundance bar plot showing the difference of relative bacterial frequency within each group for 10 different core-bacterial genera (Cutibacterium, Streptococcus, Staphylococcus, Rothia, Corynebacterium, Neisseria, Actinocymes, Haemophilus, Fusobacterium, and Veillonella). Color notation information for each genus is indicated in the footnote on the right side of the figure. **(c)** LEfSe (Linear discriminant analysis Effect Size) analysis result confirming the distinct bacterial taxonomy showing statistically significant differences in relative frequency between each group. The threshold on the logarithmic LDA (Linear Discriminant Analysis) score for discriminative features was set to 2.0 (indicating significant differential abundance).

We also conducted an integrated analysis to identify vital microbials that influence the aging group of females based on skin clinical measurement criteria and to observe changes in the microbiome by the four skin types. Using data from 750 female subjects who were distinguished into the aging groups of Young, Aging I, and Old by skin clinical measurement criteria, an alpha-diversity comparison analysis was performed. The results showed no statistically significant difference in diversity between Young and Aging I groups, but both groups showed a significant difference from the Old group (p<0.05, Kruskal-wallis) (**Figure 6a**). Among the top 10 genera, there were statistically significant differences in *Cutibacterium*, *Streptococcus*, *Rothia*, *Neisseria*, *Actinomyces*, *Haemophilus*, *Fusobacterium*, and *Veillonella* (p<0.05, Kruskal-wallis) between the aging groups (**Figure 6b**). Regarding the aging group, a LEfSe (Linear discriminant analysis Effect Size) analysis was conducted at the Genus level, and it was found that the feature microorganisms for each aging group were *Lawsonella* (Young group), *Cutibacterium* (Aging I group), and *Streptococcus* (Old group) (**Figure 6c**). All statistical values are summarized in **Supplementary Table 5**. The enterotype algorithm is a widely-used method in microbiome research that classifies the relative abundance of gut microbial communities into different types based on clustering analysis (Costea et al., 2018; Cheng and Ning, 2019). This approach has been extensively utilized to investigate the association between microbiome data and various phenotypes. However, the relationship between the skin microbiome and clinical outcomes remains an emerging field. Thus, we aimed to apply the enterotype algorithm to identify skin types of Korean women (cutotype), with the ultimate goal of deepening our understanding of the complex interplay between the skin microbiome and skin conditions.

### Strategic identification of 15 core genera via microbiome clustering and clinical correlation

**Figure 7:**
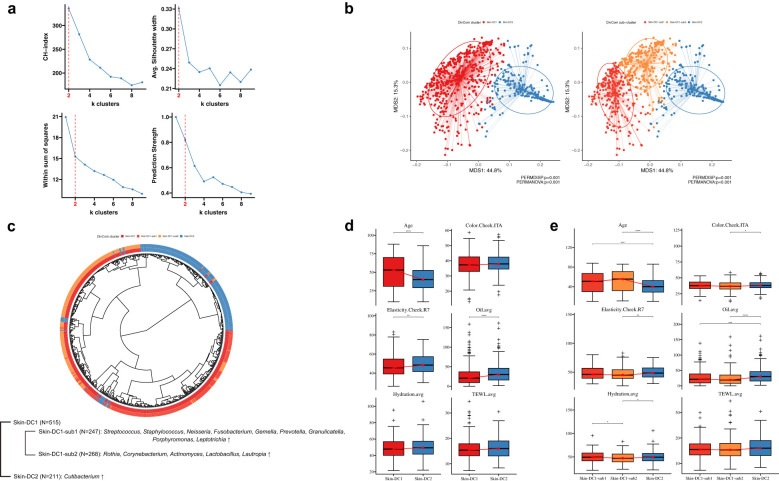
Determining KSC microbiome clusters using diversified clustering approaches. **(a)** Determine the optimal cluster with four clustering methods. The x-axis represents the number of k-clusters determined at each index, and the y-axis represents the score at the index. **(b)** Principal coordinate analysis (PCoA) indicates sample similarity based on genus-level taxonomic composition. A total of 726 subjects significantly grouped by two clusters at first. Applying the DivCom algorithm uncovered one hidden sub-cluster. The three determined clusters are labeled red (DC1-sub1), orange (DC1-sub2), and blue (DC2), respectively. **(c)** Midpoint-rooted maximum-likelihood phylogenetic tree based on taxonomy abundance of each subject. The tree contains 247 DC-sub1, 268 DC-sub2, and 211 DC2, representing genera of bacteria that are significantly enriched in each cluster. Figure **(d, e)** show the association between the two main clusters and three subclusters, respectively, and the clinical biophysics used to determine the KSC. The asterisk (*) represents the p-value of the statistical test (* < 0.05, ** < 0.01, *** < 0.001, and **** < 0.0001).

As a first step, to determine the optimal number of clusters for the Korean female skin microbiome, we implemented the Optimal Clustering method based on the Calinski-Harabasz index (CH), the Silhouette Coefficient algorithm, the within-cluster sum of squares, and the Prediction Strength. Our analysis revealed that the appropriate number of clusters was two in which divided into *Streptococcus*- and *Cutibacterium*-dominant groups in all (**Figure 7a**). Based on the top 10 genera (**Supplementary Figure 5a**; **Supplementary Table 6**), the *Streptococcus*-dominant cluster (DC1) was dominated by *Streptococcus*, *Rothia*, *Corynebacterium*, *Neisseria*, *Actinomyces*, and *Haemophilus*, while the *Cutibacterium*-dominant cluster (DC2) showed a remarkably high abundance of *Cutibacterium*. In addition, within the old group, *Streptococcus* was found to dominate the skin microbiome of women with all four clinical skin types, with an average relative abundance of 82.3%. In contrast, the Aging I and Young groups showed a relatively lower proportion, with 51.2% and 60.7%, respectively. This suggests that the abundance of *Streptococcus* in the skin microbiome may increase with age (**Supplementary Figure 5b**).

In order to further classify our samples into subgroups within two larger groups, we utilized the DivCom algorithm which identify subpopulations of microorganisms and to infer the ecological and functional roles of different taxa within the microbiome (Intze and Lagkouvardos, 2022). As a result, we were able to further divide the DC1 into two subgroups, whereas no subgroups were observed within the DC2. Among the 740 individuals satisfying the criteria for the DivCom clustering method, 726 were successfully classified into three subclusters (DC1-sub1, DC1-sub2, and DC2) using this algorithm (**Figure 7b**). Specifically, 247 individuals (DC1-sub1) within this cohort showed a high relative abundance of *Staphylococcus*, *Neisseria*, *Fusobacterium*, *Gemella*, *Prevotella*, *Granulicatella*, *Porphyromonas*, and *Leptotrichia*, with *Streptococcus*. In contrast, 268 individuals (DC1-sub2) exhibited a high relative abundance of *Rothia*, *Corynebacterium*, *Actinomyces*, *Lactobacillus*, and *Lautropia*, with *Streptococcus*. In total, 17 genera were identified as comprising 50% of the detected microbiota across all three subclusters. Comparative analysis of the relative composition of these genera across the three subclusters revealed statistically significant differences (Kruskal-Wallis p<0.05) in the relative abundance of 15 core genera. These 15 core genera were subsequently selected for downstream analysis to highlight potential associations with skin condition (**Figure 7c**). Next, we explored associations between the three groups obtained by DivCom analysis and the skin clinical measurements and compared the clinical measurements of the two groups used in the cutotype analysis (*Streptococcus* vs *Cutibacterium*) and the corresponding three groups (DC1-sub1, DC1-sub2, and DC2). The clinical metrics including age, elasticity, and oil showed differences between DC1 and DC2 groups (**Figure 7d**). Further, we also observed similar results when comparing DC1-sub1 and DC2. Interestingly, differences in skin tone between DC1-sub2 and DC2, and in moisture between DC1-sub1 and DC1-sub2 were observed (**Figure 7e**). We identified three microbial subclusters of skin based on 15 core genera and observed their associations with age, elasticity, sebum, skin color, and moisture.

### Associations of 12 KSC types within the aging group and 15 core genera

In the present study, a correlation analysis was performed to explore the association between the clinical outcomes of 15 core genera and the four KSC types within the aging group using a stepwise approach. To begin with KSC type-dependent microbiome significance within the Aging groups, yHL type in the Young group and oHL type in the Old group showed a statistically significant difference in the relative abundance of *Cutibacterium* and *Streptococcus* compared to the other three types. *Lautropica* showed a significant difference in the aLH type of the Aging I group, and *Neisseria* showed a significant difference in the oHH & oHL and oLH&oLL types of the Old group, with a clear correlation with skin tone and elasticity between the H and L type (**Supplementary Figure 6**; **Supplementary Table 7**).

**Figure 8:**
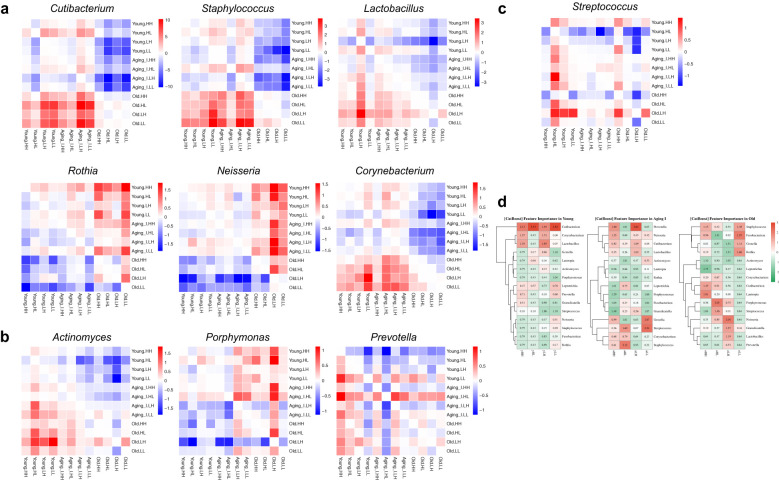
Heatmap of the Heatmap of the pairwise comparison analysis for the core genera. **(a–c)** The 10 of 15 core genera, the heatmap plots of negative log10-transformed p-values from all possible pairwise comparisons using ANCOM-BC2 were generated. Relative P-value differences between the two groups are marked with + (red) and - (blue). They all have different composition patterns, and the scale was set by their significance. The figure **(a)** shows a significant composition difference between the Young/Aging I group and the Old group, and the figure **(b)** shows that the LH and LL types in the Aging I group have the same composition pattern as the Old group. The figure **(c)** shows the specificity of certain KSCs regardless of the aging group, for example, HL in the younger group and LH in the older group with a higher composition than the other groups. **(d)** Predicting decision performance of KSCs by aging group using 15 core genera. The scale represents the z-score transformed values of the AUC score obtained from the CatBoost algorithm. The red and green colors indicate high and low feature importance, respectively.

The subsequent comparative analysis aimed to investigate the relative correlation of the 15 core genera for each of the 12 KSC types across all age groups. Using this analytical approach, we were able to confirm mosaic changes in microbial composition within the aging groups and the influence of core genera on each KSC type. We filtered genera with log_10_(-) transformed p-values exceeding ±1 based on abundance significance. Through our analysis, we observed three distinct changes in the mosaic pattern of the 15 core genera. Overall, the composition of the 15 core genera showed similar patterns between the Young and Aging I groups, but significant differences were observed between these groups and the Old group, indicating the mosaic changes (*Cutibacterium*, *Staphylococcus*, *Lactobacillus*, *Rothia*, *Corynebacterium*, *Neisseria*) (**Figure 8a**). Additionally, *Actinomyces*, *Porphymonas*, and *Prevotella* also showed high correlation between the Young and Aging I groups, but aHH&aHL types were similar to the Young group while aLH&aLL types were similar to the Old group (**Figure 8b**). These genera in the Aging I group are likely associated with skin tone based on the skin clinical measurement data. Furthermore, *Streptococcus* showed differences in all aging groups with the yHL and yLH type (**Figure 8c**). The rest of genera are also investigated the relative correlation across the 12 KSC type (**Supplementary Figure 7**).

We analyzed the microbial relative compositions that can represent each KSC type within the aging group using 15 core species, and constructed a predictive model using CatBoost boosting algorithm to distinguish the accurate KSC types within the same aging group (Prokhorenkova et al., 2018). As the microbial relative compositions differs according to the aging group, we divided the model into 80% training set and 20% test set for each group. We ran an algorithm to differentiate the microbial balance between one type and the other three types in each group. The overall validation AUC value ranges from about 0.8 to 0.98. The accuracy was about 0.96 on average in the Young and Aging I groups, but only about 0.87 on average in the Old group (**Supplementary Table 7**). The reason for the lower accuracy in the Old group is that there are minor differences that can differentiate the microbial balance in each of the four KSC types, especially in the oLL type (AUC 0.8). In the Young group, *Cutibacterium* showed a significant difference compared to the other groups and was identified as a representative species that can determine the Young group. *Lactobacillus* and *Corynebacterium* species were also relatively distinguishable in the yHH and yLH types. The Aging I group showed representative microbial balance in all four types, and interestingly, *Prevotella* in aLH type and *Streptococcus* and *Gemella* in aLL type were able to distinguish from other types. The aHL type showed a microbiological composition with a balance of *Streptococcus* and *Staphylococcus*. However, the Old group did not have enough abundance of genus composition to determine each type with a low AUC score. Simply, we observed the relative distribution of *Cutibacterium* and *Lautropia* in the oHH type, *Porphyromonas* and *Streptococcus* in the oHL type, *Nesseria* and *Granulicatella* in the oHL, and *Fusobacterium* and *Staphylococcus* in the oLL (**Figure 8d**). Based on the main 15 core microbial genera of Korean women, we identified microbial composition alterations specific to the Young and Aging I groups within three aging groups, and skin type-dependent microbiome changes in tone/elasticity and oil/moisture. The changes in core genera by aging group and skin type were demonstrated using the CatBoost boosting algorithm.

### Functional prediction of differentially enriched skin microbe by KSCs

**Figure 9:**
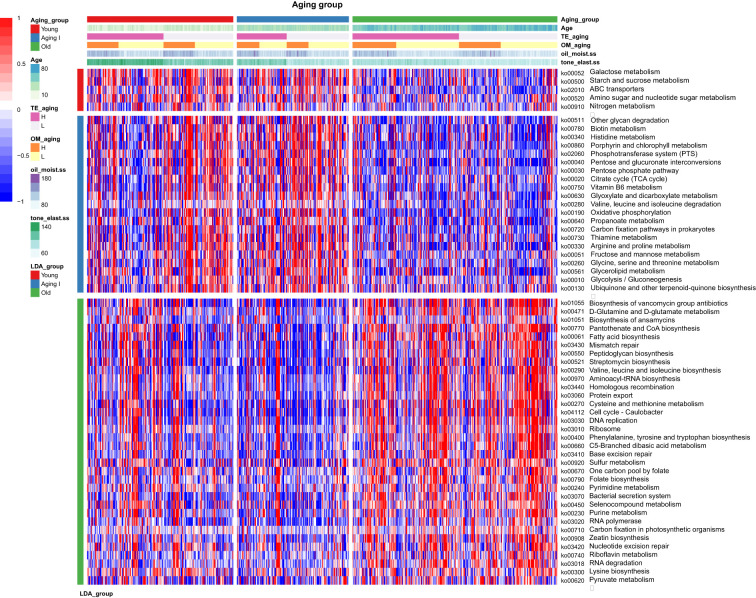
Relative abundances of the functional pathways predicted by PICRUSt2. The legend at the top provides the aging group of Korean women, their age, KSC based on Tone/elasticity, oilness/moisture, and their scores gradually. Scope and description are in the left legend. The heatmap colors with + (red) and - (blue) indicates the z-score normalized relative abundances. The young and aging groups showed contrasting functional enrichment results with the old group, and even between the young and aging groups, the young group has a distinctive enrichment pattern in KSC HH and HL types. The 59 major functional prediction of KEGG pathways are listed in detail in the **Supplementary Table 8**.

We performed PICRUSt2 analysis using the KEGG pathways (n=172) related to the KEGG orthology (n=4,837) of the 15 core genera and the feature KEGG pathways of the aging group selected through LEfSe analysis (Douglas et al., 2020). We filtered out duplicate items and pathways with an average of less than 0.5% and focused on investigating 60 pathways in detail (**Supplementary Table 8**). In the Young group, several pathways were highly expressed, including “ko00052: Galactose metabolism”, “ko00500: Starch and sucrose metabolism”, “ko02010: ABC transporters”, “ko00520: Amino sugar and nucleotide sugar metabolism”, and “ko00910: Nitrogen metabolism”. Additionally, in the Young group, the L- type showed a relatively higher pattern in the Tone/elasticity criteria compared to the H- type. Specifically, the H- type in the Oil/Moisture criteria showed a higher pattern in the -L type, which was highlighted in the yLH group with a high expression of pathways. In the Aging I group, 21 pathways showed high enrichment compared to other groups. Interestingly, the functional pathways in Aging I group showed a similarity to the L- type in tone/elasticity criteria of the young group. However, the L- type showed a higher abundance than the H- type in tone/elasticity criteria in Aging I group. The -H type and -L type samples in oil/moisture criteria showed a relatively low abundacne in these KEGG pathways, which mainly belonged to carbohydrate metabolism, amino acid metabolism, and metabolism of cofactors and vitamin categories. Finally, in the Old group, interestingly, pathways related to genetic information processing showed high abundance, such as “ko03430:Mismatch repair”, “ko03440:Homologous recombination”, “ko03410:Base excision repair”, “ko03020:RNA polymerase”, “ko03420:Nucleotide excision repair”, and “ko03018:RNA degradation”. In addition, the KEGG pathway enrichment results specific to the Old group were very different from those of the Young and Aging I groups, with a very predominant pattern. This indicates that the predicted functional pathway patterns of the Young and Aging I groups are more similar to each other than to the Old group (**Figure 9**).

## Discussion

In the dermatology and cosmetics field, the Baumann Skin Type Indicator (BSTI) has been widely used to classify facial skin types based on a questionnaire (Baumann, 2008; Lee et al., 2017, 2019). In Korean women, OSNT, DSNT, DRNT, and OSNW have been reported as the most common skin types (Ahn et al., 2017). In this study, we first selected 60 questions from the existing 77 Baumann questionnaire types and the microbiome questionnaire through correlation analysis with biophysical clinical indicators of the skin (**Supplementary Table 1**). We then determined the Baumann skin types for a total of 792 women using the Baumann questionnaire items. The top four groups accounted for 66% of the participants, with the order being DRNT, DRNW, DSNT, and OSNT. However, the results were different from those of previous studies. In the case of Korean women, most tend to choose dry skin type rather than oily skin type, and the majority of women tend to choose non-pigmented options. The conventional method of defining skin types based on questionnaires is quite subjective and may not provide objective results. Thus, our study reaffirms the importance of defining skin types using more objective indicators.

To compensate for this and derive objective indicators, various biophysical skin clinical measurement devices are used to measure skin conditions (Stettler et al., 2021; John et al., 2023). However, measurement parameters may have bias due to the environment at the time of measurement and deviations in the parameters. Through the process of minimizing the number of variables, we systematically selected parameters that can represent multivariate parameters. To eliminate the bias of the 39 skin measurement data, we minimized the correlation between the measurement results and the deviation of the skin measurement sites, resulting in the determination of six skin clinical metrics. Interestingly, when predicting age groups using all 39 clinical parameters through machine learning, the performance (micro-average ROC-AUC score) was 98%, six clinical metrics also accomplished around 90% accuracy (highest = 0.94) (**Figure 2**). Although there was a difference in performance compared to the prediction using 39 clinical indicators, the compact six clinical indicators are suitable factors for distinguishing age groups and suggest that skin condition can be well represented by objective factors such as oiliness, moisture, tone, elasticity, wrinkles, and pores. Among the six clinical metrics, wrinkle and pore metrics had a high correlation with color and elasticity, so we were able to define the aging group-specific skin types of Koreans participating in this study using only four criteria. The four skin indicators derived through this study can be categorized into color/elasticity and oil/moisture, with the values being High (H) or Low (L) depending on the level of each indicator. The combinations of these indicators can be evaluated as HH, H/L, L/H, and L/L groups. The prefix H or L represents high or low color and elasticity of the skin, while the suffix H or L represents the low state of oil and moisture. Additionally, we confirmed that the Yong group (10–34 years old) was differentiated by facial color tone and skin elasticity criteria (Young), the Aging 1 group (35–50 years old), and the Old group (51 years old and above) were differentiated by oil criteria. In another study involving 100 Korean women, it was reported that even with 10 skin clinical indicators, the clinical parameters of wrinkle, pigmentation, and elasticity were more decisive factors for aging than the oil and moisture (Cho et al., 2019). This finding is consistent with our observations, suggesting that even with numerous skin clinical indicator parameters, the clinical indicators that can determine skin aging can converge to a smaller set of critical factors. Through this study, it is evident that skin tone and elasticity are the most important indicators for distinguishing between aging groups. As we move from the Young to the Old group, the Tone and Elasticity gradually decreases. In conclusion, skin tone and elasticity showed distinct correlated changes with skin aging as age increased, while oil and moisture levels were determined more by individual differences than by age (**Figure 5**). This finding highlights the importance of considering both the common age-related factors (tone and elasticity) and the individual factors (oil and moisture levels) when addressing skin aging concerns and developing personalized skincare strategies. By taking into account these different aspects, more effective and tailored solutions can be provided to individuals with varying skin types.

Various host factors, including gender, age, and ethnicity, influence the composition of skin microbial communities, which also varies across different regional factors such as temperature, humidity, and exposure status. These both factors play a crucial role in the growth and maintenance of the resident microbial population on the skin (Fierer et al., 2008; Ying et al., 2015). Indeed, the interactions between microbes and these variabilities, as well as with other microbes in the same region, are potentially site-specific in faces. In our study, the analysis of the facial skin microbiome involved 950 clinical participants (756 females and 194 males), using facial wash water that could represent the entire face, thereby eliminating region-specific differences such as the forehead, cheeks, or nose (Lee et al., 2021).

To focus on the differences in the distribution of skin microbes between female and male, the alpha diversity of the microbiome was found to be higher in females than in males, with a particularly significant difference observed in individuals aged 50 and above. In both genders, statistically significant distinctions were observed in five microbial genera (*Cutibacterium*, *Streptococcus*, *Staphylococcus*, *Rothia*, and *Neisseria*). Especially, *Staphylococcus* levels decreased with age in females, while they increased with age in males. Our study is the first large-scale comparison of the skin microbiome between female and male Korean participants. Considering the limited research comparing genders across different ethnicities, our findings suggest that there is a need for further comprehensive, gender-specific research in this area (Leung et al., 2015). In recent studies targeting Chinese participants aged 25–35 as younger type and those aged 56–63 as aging type, as well as Korean participants aged 19–28 as younger type group and those aged 60–63 as aging type, there was a somewhat biased tendency in the diversity of the age spectrum by excluding phenotypes based on objective skin clinical indicators (Kim et al., 2022). Additionally, there was a clear limitation in the number of recruited participants. In our study, we conducted the first-ever association analysis between the skin microbiome and three aging groups and four skin types per group, determined based on sample homogeneity, uniformity of age spectrum, and various objective skin clinical measurement indicators.

Using the enterotype algorithm, we examined the skin types of Korean women and found that their types were predominantly characterized by *Streptococcus* and *Cutibacterium*. Considering the Chinese study that *Moraxella* and *Cutibacterium* were representative skin types or cutotypes, we confirmed the existence of unique cutotypes due to racial or geographical differences, suggesting the need for tailored care according to skin types (Li et al., 2021). Furturemore, a stark difference in skin microbiome between Central Africans and East Asians can be observed, with Cameroonians being dominated by the *Micrococcus* type and Japanese individuals being dominated by the *Cutibacterium* type (Ogai et al., 2022). *Cutibacterium* species (*C. avidum*, *C. acnes*, and *C. granulosum*) colonize the skin of healthy individuals and acne patients, possibly due to their affinity for sebum-rich areas of the skin. The abundance of *Cutibacterium* in Koreans, Chinese, and Japanese individuals suggests that the presence of more sebum in East Asians provides many benefits for the growth of *Cutibacterium* on the skin surface or sebaceous glands (Nordstrom and Noble, 1984; Vashi et al., 2016).

We went beyond the existing enterotype algorithm and utilized the DivCom algorithm to define a total of 3 groups, including subgroups (DC1-sub1, DC1-sub2, DC2). In these 3 groups, 15 genera with a detection rate of 50% among the total sample count (Kruskal-Wallis p <0.05) were selected as core microbe (**Figure 7**). The skin clinical measurement data and microbial clustering analysis revealed significant differences in age, elasticity, and sebum levels between the DC1 and DC2 groups. Additionally, the clustering of DC1-sub1 and DC1-sub2 was closely related to the level of moisture. This result for DC2 is consistent with a previous study showing an increasing trend in sebum-related clinical skin measurements in the cutotype dominated by *Cutibacterium* (Li et al., 2021). *Cutibacterium* spp., such as *C. acnes*, typically hydrolyze triglycerides in sebum to release free fatty acids, which contribute to sebaceous gland clustering, and their accumulation influences the acidic pH (~5) of the skin surface (Marples et al., 1971; Gribbon et al., 1993). Considering that the cutotype clusters of DC1 and DC2 have distinct strategies, many common pathogenic microbes, such as *Streptococcus* spp. (*e.g. S. pyogenes*) and *Staphylococcus* spp. (*S. aureus*), are also known to be inhibited by the acidic pH strategy (Bomar et al., 2016). Therefore, it is estimated that in women with low sebum levels and reduced lipid accumulation, the distribution of *Cutibacterium* spp. decreases, resulting in the increased detection of pathogenic bacteria such as *S. pyogenes* and *S. aureus*. *Neisseria*, *Fusobacterium*, *Gemella*, *Prevotella*, *Granulicatella*, *Porphyromonas*, and *Leptotrichia* showed significant abundance in the DC1-Sub1 cluster, while *Rothia*, *Corynebacterium*, *Actinomyces*, *Lactobacillus*, and *Lautropia* showed significant abundance in the DC1-Sub2 cluster. A higher moisture level in the skin might create a more favorable environment for certain bacteria, such as those found in the DC1-Sub1 cluster, while drier conditions might favor the growth of bacteria in the DC1-Sub2 cluster. As the interactions and mechanisms of symbiosis between coexisting microorganisms in the skin environment are still poorly understood, it is necessary to study the skin microbiota of different ethnicities or diseases to better understand the causes and pathogenesis of skin diseases and to develop strategies for prevention and treatment of skin diseases (Lemoine et al., 2020; Loomis et al., 2021).

Further, based on the four KSC types of aging groups, a survey was conducted on the normal distribution of 15 core skin microorganisms. As a result, among the 15 core genera, *Cutibacterium*, *Staphylococcus*, *Lactobacillus*, *Corynebacterium, Rothia*, and *Neisseria* showed distinct microbial cluster differences between young and aging I groups vs the old group, confirming the differential composition of specific-skin types. These results show that skin microbiome patterns vary slightly from person to person based on region and nationality - Egyptians have more bacteria from the phylum *Pseudomonadota*, Cameroonians (*Staphylococcus* and *Micrococcus*), South Asians (*Corynebacterium* and *Streptococcus*), and Japanese (*Cutibacterium*) - but even Korean skin has a characteristic distribution of the same categories of microbes across age groups (Ramadan et al., 2016; Chaudhari et al., 2020; Cho and Eom, 2021; Ogai et al., 2022). Especially in our skin type, *Cutibacterium* and *Staphylococcus* are shown little less composition in yHL and oHL skin types while *Streptococcus* is higher in yHL type. These results indicate that *Cutibacterium*, a lipopolyic bacterium preferentially present on sebaceous skin sites, is closely involved in the Young and Old groups, in contrast to the notion that the ratio of *Cutibacterium* to *Staphylococcus* and *Streptococcus* varies depending on the oil/moisture skin environment, or vice versa (Conwill et al., 2022). In addition, the young group includes puberty, which brings significant changes to the skin microbiome, such as I) hair growth and hair pattern are promoted by androgens, II) sebaceous gland development and increased sebum production, III) apocrine gland development, IV) skin development through estrogen and progesterone (Gribbon et al., 1993; Beier et al., 2005; Wilkinson and Hardman, 2017; Gratton et al., 2022). According to the intrinsic physiological regulation of hormones by the sexual maturation process, the prominent changes in skin microbiota from *Cutibacterium* to *Staphylococcus* and *Streptococcus*, as well as *Lactobacillus* (higher in yLH), *Actinomyces* (higher in yHL), *Porphyromonas* (lower in yHL), and *Prevotella* (lower in yLL), were predictable. Even after the turbulent period of skin development such as puberty, *Cutibacterium*, *Staphylococcus* and *Streptococcus* were found to have skin type-specific microbial composition, with *Actinomyce* and *Porphyromonassms* showing opposite microbial composition patterns within aHH/aHL and aLH/aLL. These results suggest that the alteration in the abundance of these two microorganisms in the aging I group is an important factor in determining skin tone/elasticity. As per the Old group, most microbiota except *Neisseria* showed insufficient association to determine skin type specificity within the Old group. This suggests that the decrease in elasticity due to changes in the thickness of the skin layer (dermis and epidermis) and the decrease in the activity of sebaceous glands and sweat glands, which can occur with aging, potentially affect the growth and survival of various types of bacteria in the skin, causing changes in the skin microbiota balance (Ratanapokasatit et al., 2022). However, Neisseria showed a significant increase in composition in oLH and oLL skin types, which is consistent with the results of prioritization of *Neisseria* in dry skin group in Chinese women (Zheng et al., 2021).

CatBoost is a machine learning algorithm designed to process categorical data, which is data organized into categories or labels, and is a suitable technique for typing analysis to process high-dimensional data with many categorical features, such as skin microbiome data, to identify and classify skin microbes by clinical skin type (Prokhorenkova et al., 2018; Han et al., 2022). Therefore, we used CatBoost to analyze 15 core genera to represent each KSC type in each aging group and constructed a prediction model to distinguish them from the other three types within the same aging group. Overall, the validation AUC value ranged from about 0.8 to 0.98. The Young & Aging I group showed a high accuracy prediction process with an average of about 0.96, but the Old group showed a relatively low prediction rate with an average of about 0.87. The reason for the lower accuracy in the Old group was the lack of significantly classifiable microbiota in the four KSC types, which resulted in a low prediction rate due to similar abundance across the board. In the Old group, there is a general reduction in microbial diversity and increased dominance of *Streptococcus* across all skin types (**Figures 7**, **8**). We assumed that this convergence leads to less distinguishable microbial features between KSC types, reducing the discriminative power of the model.

Through typing analysis utilizing the CatBoost algorithm, we determined that *Cutibacterium* was the preferred indicator in the Young group compared to the rest of the groups and coexisted with *Corynebacterium* and *Lactobacillus* in the yHH and yLH types with high oil/moisture indicators. On the other hand, *Cutibacterium* was predicted as the main feature in yHL and yLL types. This result suggests that *Cutibacterium*, *Corynebacterium*, and *Lactobacillus* microbiota are important factors in determining skin condition in the younger group due to continuous changes in the skin environment and growths. In the Aging I group, we found a balanced microbial distribution of *Cutibacterium*, *Streptococcus*, *Staphylococcus*, *Corynebacterim*, and *Nesseria*, which are representative of the normal skin microbiota in the aHH group. Maintaining a balanced skin microbiome is crucial for skin health. An imbalanced skin microbiome, with an overgrowth of certain types of bacteria or a decrease in beneficial bacteria, can potentially contribute to the development of various skin conditions and diseases. This is because the skin microbiome plays a vital role in maintaining skin health by protecting the skin from harmful pathogens, regulating the immune system, and contributing to the maintenance of skin hydration and pH (Byrd et al., 2018; Carmona-Cruz et al., 2022). In addition, the aHL type was specifically enriched with *Streptococcus*, *Staphylococcus*, and *Corynebacterim*, the aLH (*Prevotella*), and the aLL (*Streptococcus* and *Gemella*). The Old group did not have the abundance of strains to determine each type with a low AUC score, but interestingly, the oHH and oHL types, the KSC types that maintain high elasticity despite aging, still showed a *dominance* of *Cutibacterium* and *Corynebacterim*, while the oLH and oLL types lost the balance of normal skin microbiota. *Streptococcus*, *Staphylococcus*, *Corynebacterium*, *Roseomonas*, and *Micrococcus*, among others, are known to influence gene expression and mechanisms that determine the condition of the skin, such as differentiation and proliferation of skin cells, and barrier formation (Vashi et al., 2016). Therefore, analyzing the correlation between skin microbiome and the physical indicators of skin condition can provide important foundational data for the development of personalized cosmetics, as well as potentially serving as a novel tool and target in the fields of medicine, pharmacy, and cosmetics. The 15 core genera and KSC-based stratification framework may serve as actionable biomarkers for non-invasive diagnostics and tailored dermocosmetic interventions targeting age- and phenotype-specific skin conditions. These insights provide a foundation for precision skincare and predictive skin health monitoring. In order to address unresolved issues such as race, environmental factors, hormonal status, and geographical differences, efforts are needed to advance standardized skin clinical measurement and skin microbiome analysis methods for the realization of “The Skin Microbiome Atlas”. Training a model that accurately classifies individuals based on their skin microbiome and skin measurement data, using CatBoost machine learning based on race, country, and gender, has the potential to be a tool that can be applied in the field of forensics to identify the race and geographical location of perpetrators or victims of crimes or disasters, as well as for the treatment of skin diseases mediated by microorganisms, the development of cosmetics, and the advancement of preventive medicine in the field of skin health (Farberg et al., 2020; Boxberger et al., 2021; Cho and Eom, 2021; Habeebuddin et al., 2022; Carvalho et al., 2023).

Our study aimed to examine the ideal microbiome-biophysical association of the skin and identify skin types based on skin measurement parameters and microbiome association. The researchers established quantitative data of multiple parameters, including skin tests, clinical surveys, and skin microbiome of 950 Korean subjects (756 female and 194 male). Based on statistical analysis and cross-validation of machine learning - xgboost with 39 skin parameters, six parameters were integrated, differentiating four representative criteria (tone/elasticity and oil/moisture). We created four KSCs using four parameters: tone, elasticity, oil, and moisture. Furthermore, based on the distribution of all age groups according to the four-skin biophysical parameters, we identified three aging groups (Young (under 34 years old), the Aging I group (35-50), and the Old group (over 51)). Tone/elasticity plays an important role in dividing the aging groups, while oiliness/moisture plays an important role in distinguishing individual differences within the aging groups. To analyze the correlation between skin microbiome and KSC types, microbiome clusters and dominant bacterial genus according to each skin phenotype and aging group were ferreted out. Through DivCom clustering analysis, 726 female skin microbiomes out of 740 were successfully divided into three subclusters (DC1-sub1, DC1-sub2, and DC2), and 15 core genera, including *Streptococcus* and *Cutibacterium* as the bimodal center of the clusters, were identified as the key microbiota that determine the skin condition and skin microbial environment. The utilization of the CatBoost boosting algorithm helped to identify skin microbiota with distinguishing power based on the KSC type within each aging group. The study demonstrated that 15 core genera can be used as objective indicators to differentiate the microbial composition between the Young, Aging I, and Old groups. The validation AUC value showed a high average accuracy of 0.96, sufficient for predicting skin types based on microbial composition in the Young and Aging I groups. Our microbiome-biophysical association study is expected to have significant practical applications in various fields, including prevention and treatment of skin diseases, development of cosmetics, microbial and skin immune cascade, and biomaterials derived from microorganisms, in the analysis of the skin microbiome.

## Data Availability

The dataset presented in this study can be found in the online repository. The repository name and access number can be found in the Availability of data and materials in Materials and Methods.
