# Supplementary material for: Skin microbiome-biophysical association: a first integrative approach to classifying Korean skin types and aging groups
Source: Front Cell Infect Microbiol. 2025 Jul 7;15:1561590. doi: 10.3389/fcimb.2025.1561590 (PMC12277360; doi:10.3389/fcimb.2025.1561590)
Supplement: Supplementary file 1 [file DataSheet1.pdf]

Supplementary Material 1. Detailed Methods of Biophysical Measurement for Skin Conditions

Measurement of skin hydration

In this study, epidermal skin hydration of the facial area (forehead and cheek) was measured thrice using Corneometer® CM 825 (C+K, Germany) at baseline, and the average value was analyzed. The epidermis has the characteristic of having a high resistance to electricity. All electrical phenomena are caused by the movement of charges, and capacitance refers to the ability to store these moving charges. In this device, an electric field is formed between the electrode plates of the probe, and the moisture content in the stratum corneum layer of the epidermis can be measured based on the capacitance generated at this time.

Measurement of Transepidermal Water Loss (TEWL)

The amount of transdermal water loss (TEWL) at each test site (forehead and cheek) was measured three times using the Tewameter® TM HEX (C+K, Germany) at baseline, and the average value of the stabilization section was analyzed. This device is an open chamber type with the top and bottom of the probe open, and moisture does not accumulate in the probe, allowing long-term measurement. The temperature and humidity sensor inside the probe measures the amount of water evaporation (g/h/m2) over time per unit area of the test site.

Measurement the amount of skin sebum

The amount of skin sebum at the forehead, nose, left and right cheek sites was measured using the Sebumeter® SM815 (C+K, Germany) at baseline, and the average value of the stabilization section was analyzed. This device measures the amount of sebum per unit area (㎍/㎠) based on the principle of light reflection by contacting a translucent sebumeter cassette with the skin for about 30 seconds.

Measurement of skin wrinkles (Lateral canthal lines and Nasolabial)

3D image of the selected lateral canthal lines (eyes) and nasolabial (nose) was photographed using PRIMOSCR (Canfield, USA) at baseline. Parameters of the skin wrinkles (Ra value) were analyzed by PRIMOSCR Software.

Measurement of the skin pore

At baseline, the facial image for the cheek was taken in optical mode using Mark-Vu (Mark-Vu®; PSI PLUS Co., Ltd., Suwon, Korea). The photographed optical image was analyzed by measuring the percentage (%) of the pore spot area for the analysis area of the cheek area using Image-Pro® 10 (Media Cybernetics, USA).

Measurement of skin color and skin tone

The skin color (lightness (L*), greenness (a*), and yellowness (b*)) and skin tone (ITA° value: Individual Typology Angle) of the left or right cheek area was measured three times at baseline using Spectrophotometer® CM26dG (Minolta, Japan), and the average value was analyzed. The optical system of this device follows the colorimetric system of CIE L*a*b* by measuring the spectral reflectance and tristimulus values of the sample using the diffuse illumination 8° light receiving method equipped with the integrating sphere.

Measurement of skin elasticity

In this study, the skin of the left or right cheek area was measured using the Cutometer® MPA580 (C+K, Germany) at baseline. Skin elasticity was analyzed by the R2 parameter (Ua/Uf; gross elasticity or overall elasticity of the skin), R5 (Ur/Ue, the net elasticity), and R7, (Ur/Uf , the ratio of elastic recovery to the total deformation). This device measures and shows skin elasticity in graphs and values by absorbing the skin for 2 seconds 3 times in a row at a constant negative pressure of 450 mbar.

Measurement of dermal density

At baseline, the dermal skin density of the left or right cheek area was measured using the Ultrasound Probe of DermaLab® Series SkinLab Combo (Cortex Technology, Denmark). This device is based on an ultrasonic system intended to be used to visualize the layers of the skin.

Supplementary Material 2. Determination of four skin criteria (tone/elasticity and oil/moisture) analysis values from skin biophysical parameters

The z-score, also known as Standard Scalar, is a method designed to adjust scores to a common scale for comparison, with a mean of 100 and a standard deviation of 20. It is used as a relative measure to assess how well a score compares to others. The equation for calculating the SS(x) from the raw score (x) with a mean of ($x$mean) and a standard deviation of (std) is:

SS Function: Standard Scalar (mean=100, std=20)

$$SS(x) = \frac{20 \times (x - x_{mean})}{x_{std}} + 100$$

The z-score allows for comparing scores from different distributions on a standardized scale, providing insight into how well a score performed relative to the mean and variability of the data.

Tone and Elasticity value (TE_{ss})

$$TE_{ss} = SS(Color.ITA_{ss} \times Elasticity.R7_{ss}) = SS\{SS(Color.Cheek.ITA) \times SS(Elasticity.Cheek.R7)\}$$

Oil and Moist value (OM_{ss})

$$OM_{ss} = SS(Oil_{ss} \times Moist_{ss}) = SS\{SS(Oil_{mean}) \times SS(Moist)\} = SS\left\{SS(Oil_{mean}) \times SS(\frac{Hydration_{mean}}{TEWL_{mean}})\right\}$$

$$Oil_{mean} = \frac{Oil.Forehead + Oil.Nose + Oil.Cheek}{3}, Hydration_{mean} = \frac{Hydration.Forehead_{mean} + Hydration.Cheek_{mean}}{2}, TEWL_{mean} = \frac{TEWL.Forehead_{mean} + TEWL.Cheek_{mean}}{2}$$

Good Moist Condition: TEWL Low & Hydration High
The term "Moist" is defined as the average value of hydration divided by the average value of Transepidermal Water Loss (TEWL).

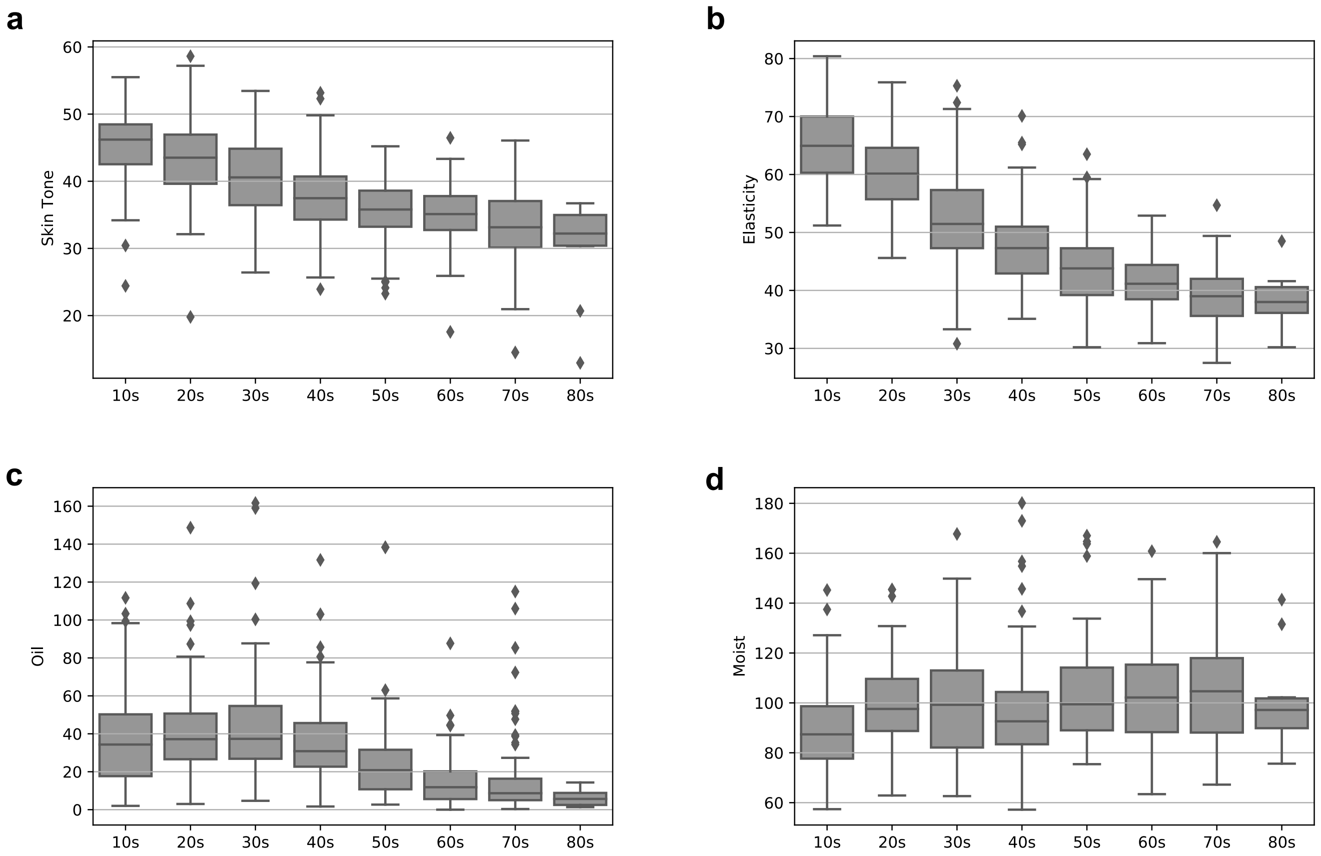

Supplementary Fig. 1. Distribution of **(a)** tone, **(b)** elasticity, **(c)** oiliness, and **(d)** moisture biophysical parameter values by age. The y-axis of the quantile box plot represents the range of results for each biophysical parameter, and the x-axis represents the age in 10-year increments. The diamond symbol is an outlier.

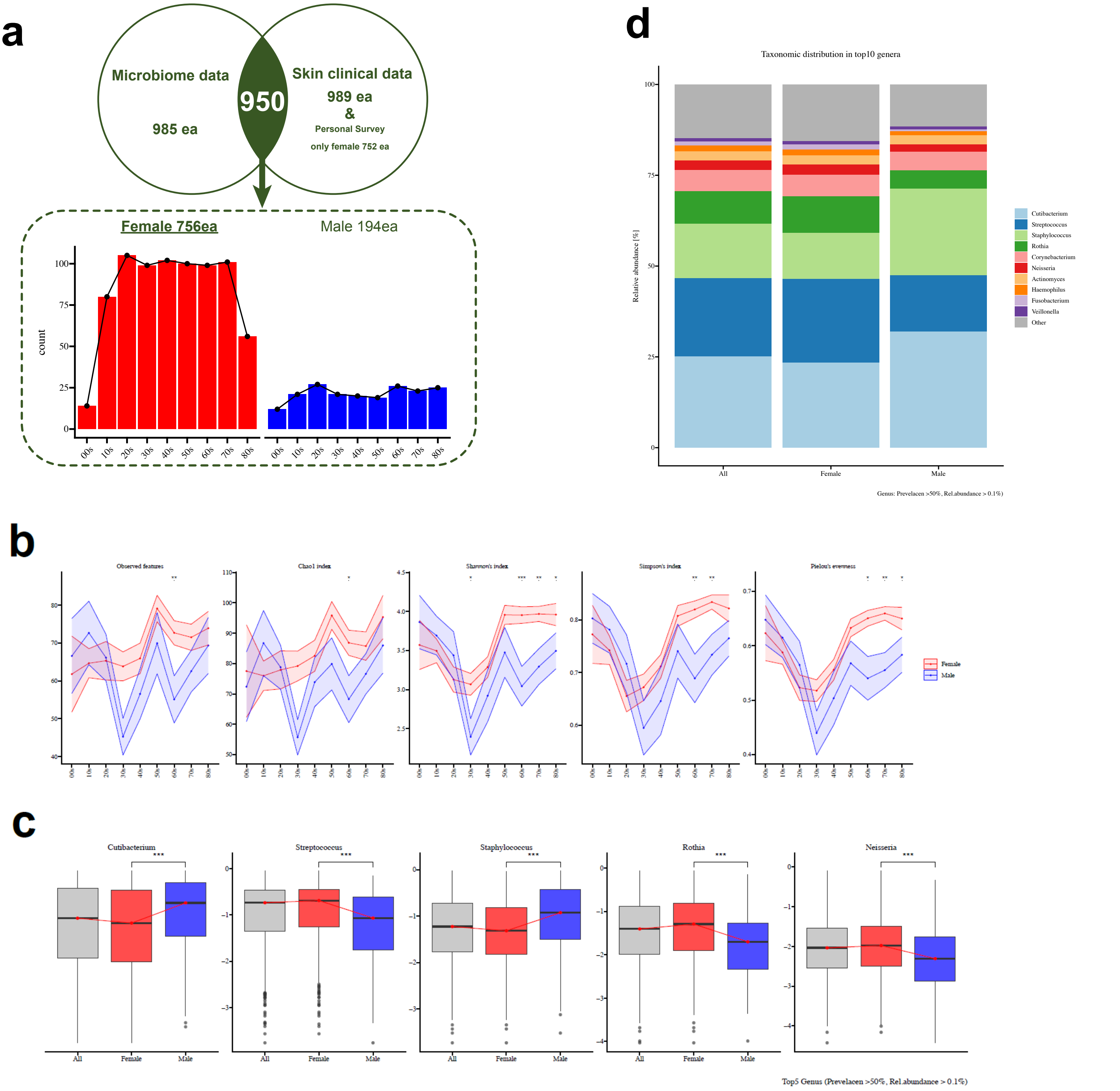

Supplementary Fig. 2. Korean skin microbiome configuration wave.
(a) Number and age distribution of samples used to determine Korean Skin Cutoype (KSC). The number of subjects in the overlapping areas of the Venn diagram indicates that skin clinical biophysics data is accompanied by microbiome data. Minimized margin of error for age indicates a homogeneous age sample population. (b) Graph of wave lines showing estimated microbial alpha-diversity results by sex and each age group (F: Female, was indicated red line color; M: Male, was indicated blue line color). The Observed features, Chao 1, Shannon, Simpson, and Pielou_e alpha-diversity indices were applied to this alpha-diversity estimation, and it is a measure for confirming the microbial richness and evenness in each group. The asterisk (*) represents the p-value of the statistical test (** < 0.01). (c) Box plot showing statistically significant relative microbial frequency differences for six representative bacterial genera (Cutibacterium, Streptococcus, Staphylococcus, Rothia, Corynebacterium, and Neisseria) between each comparison group (all: all donors, was indicated gray color; F: Female, was indicated powder pink color; M: Male, was indicated sky-blue color). (d) Relative abundance bar plot showing the difference of relative bacterial frequency within each group (all: all donors group; F: Female group; M: Male group) for 10 different core-bacterial genera (Cutibacterium, Streptococcus, Staphylococcus, Rothia, Corynebacterium, Actinocyces, Neisseria, Haemophilus, Fusobacterium, and Veillonella). Color notation information for each genus is indicated in the footnote on the right side of the figure.

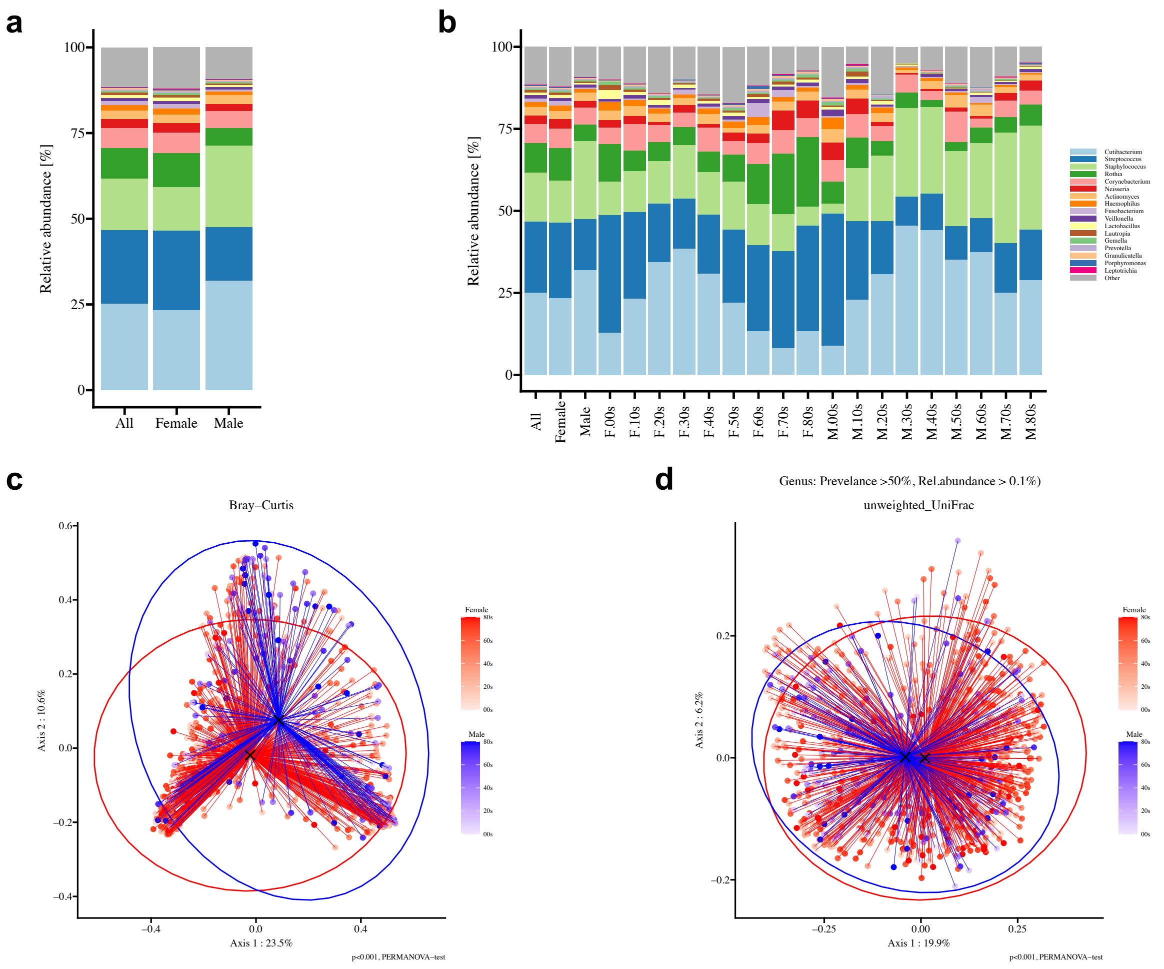

Supplementary Fig. 3. Comparison of bacterial relative frequency and compositional difference (beta-diversity) between each sex group. **(a)** Relative abundance bar plot showing the difference of relative frequency of the classified bacterial genera within each group. **(b)** Relative abundance bar plot showing the difference in relative bacterial frequency for each age group according to different sex groups. **(c, d)** Principal Coordinate Analysis (PCoA) plots show the bacterial compositional differences (beta-diversity) between two different sex groups. Bray-Curtis (considering microbial abundance) and unweighted_UniFrac (considering phylogeny) distance dissimilarity estimation matrices were applied to these Beta-diversity analyses.

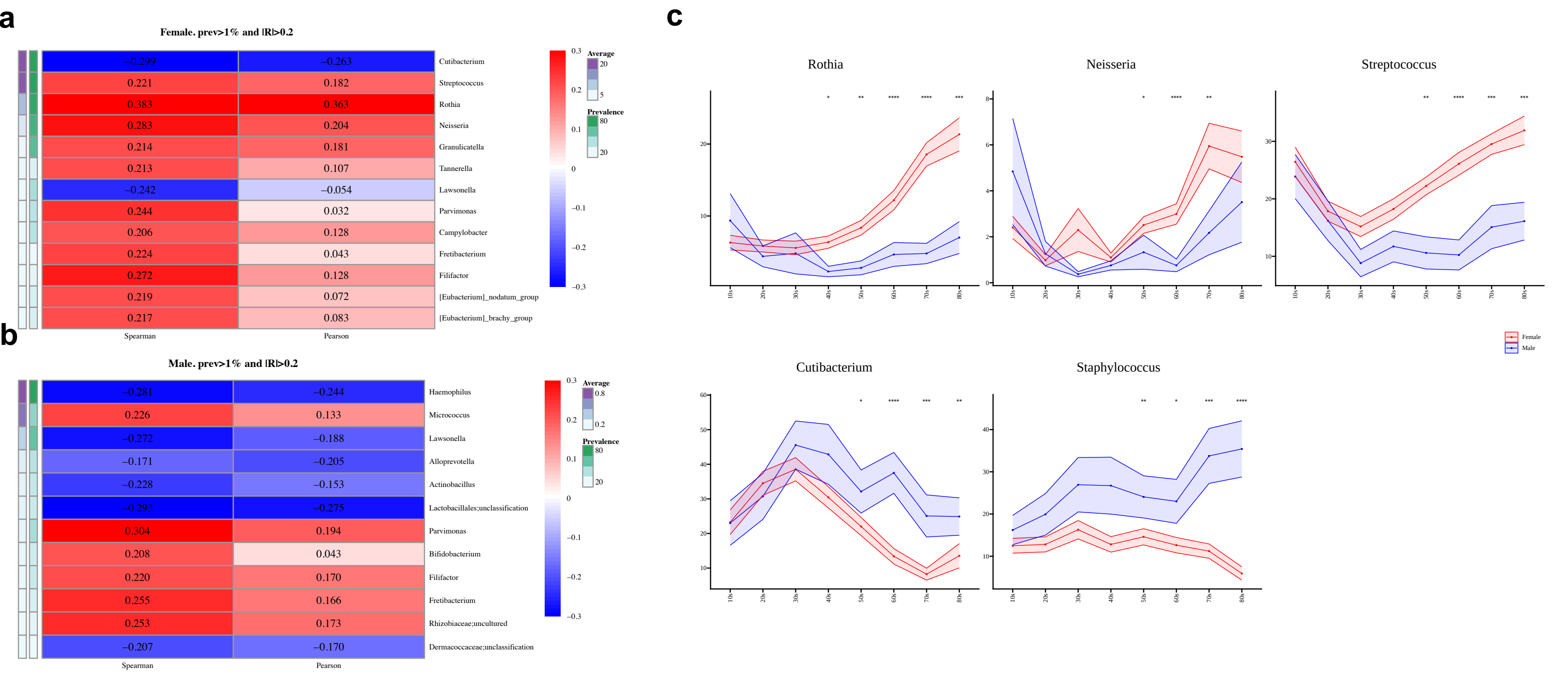

Supplementary Fig. 4. Correlation analysis between age and sex groups for the significant skin microbiome. **(a, b)** Heatmap plots showing the correlation between sex and age for each skin microbiome. Spearman and Pearson correlation analysis were applied to association prediction. These plots provide a measure to predict the association of each significant skin bacterial genus with aging according to different sex groups. **(c)** Graph of wave lines showing the difference of relative bacterial frequency by age group for each sex group (F: Female, was indicated red line color; M: Male, was indicated blue line color) for five different core bacterial genera (*Rothia*, *Neisseria*, *Streptococcus*, *Cutibacterium*, and *Staphylococcus*). The asterisk (*) represents the p-value of the statistical test (* < 0.05, ** < 0.01, and *** < 0.001).

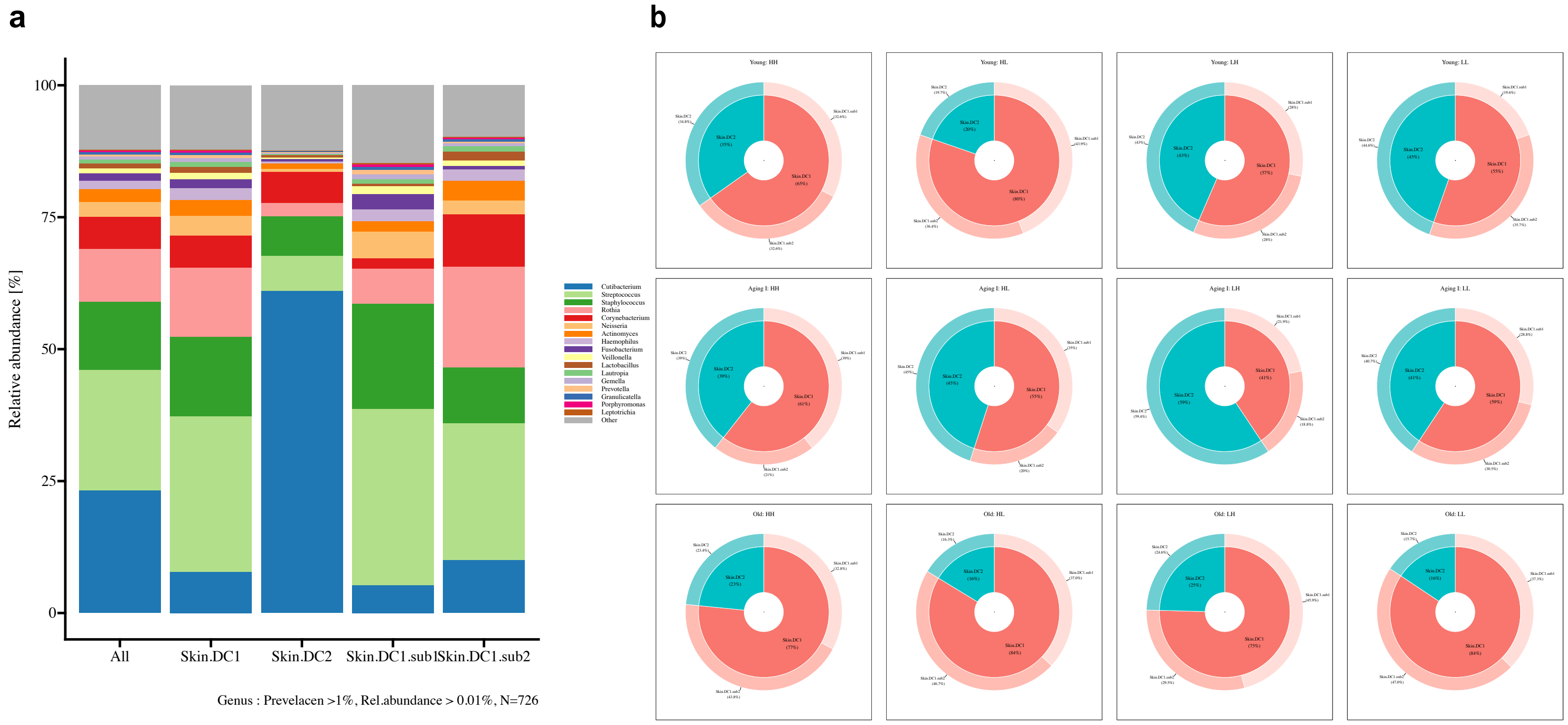

Supplementary Fig. 5. Investigation of the association between the Aging groups and KSCs depending on bacterial dominant clusters (DCs). **(a)** Bar plot showing clustering result for dominant bacterial clusters based on the relative frequency of top 10 core bacterial genera (*Cutibacterium*, *Streptococcus*, *Staphylococcus*, *Rothia*, *Corynebacterium*, *Neisseria*, *Actinomyces*, *Rhodopseudomonas*, *Haemophilus*, and *Fusobacterium*) within each sample. The two optimal cluster numbers were calculated using the DivCom clustering method with the Calinski-Harabasz index (CH) and the Silhouette Coefficient algorithm. **(b)** A pie chart showing the distribution of donors included in two dominant bacterial clusters within KSC types according to each age group (S-cluster: *Streptococcus*-dominant cluster, was indicated powder pink color; C-cluster: *Cutibacterium*-dominant cluster, was the sky-blue color). Pie charts in the out layer also include sub-clusters.

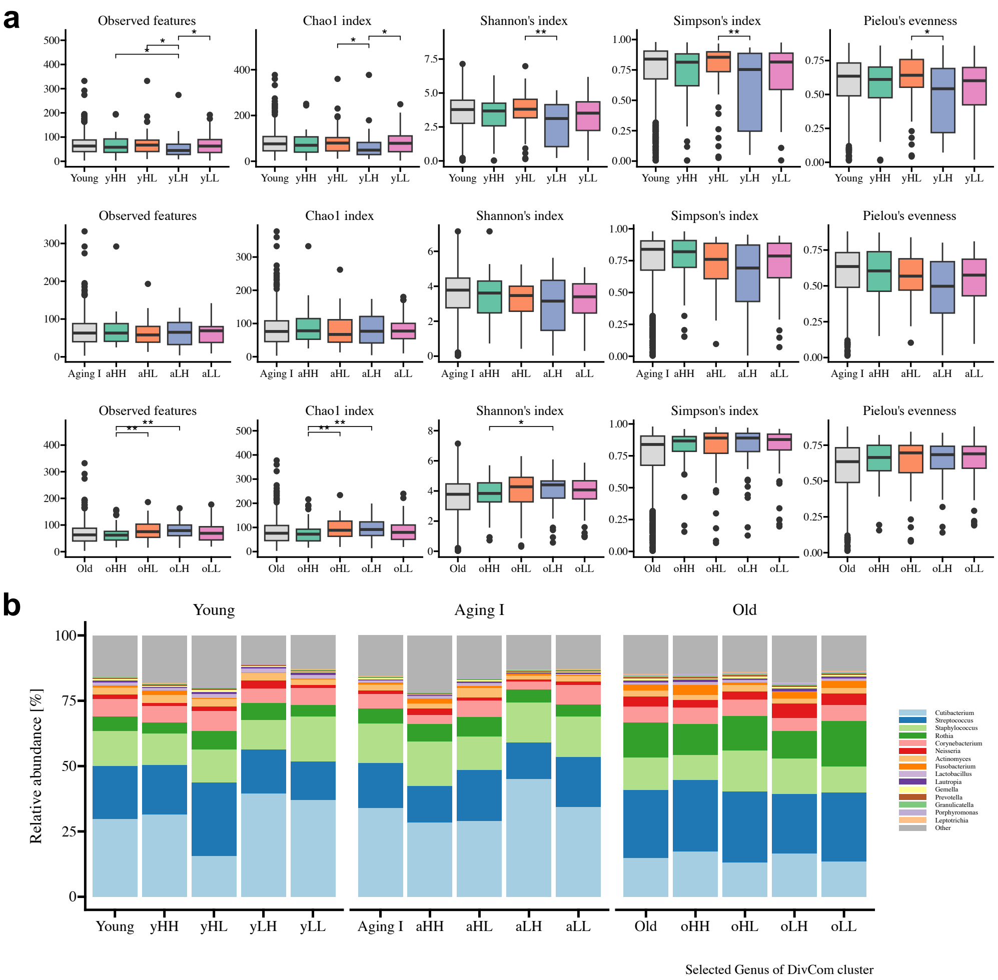

Supplementary Fig. 6. Comparison of relative frequency within each KSC type for 15 core skin microbiomes identified by DivCom clustering method. **(a)** Box plot showing microbial alpha-diversity comparisons for the four KSCs of each Aging group. The five-representative alpha-diversity indices were applied to this alpha-diversity estimation, and it is a measure for confirming the microbial richness and evenness in each group ('p' means the p-value). The asterisk (*) represents the p-value of the statistical test (* < 0.05 and ** < 0.01). **(b)** Relative abundance bar plots show the relative distribution rate within KSC types by each age group for 15 core bacterial genera (*Cutibacterium*, *Streptococcus*, *Staphylococcus*, *Rothia*, *Corynebacterium*, *Neisseria*, *Actinomyces*, *Fusobacterium*, *Lactobacillus*, *Lautropia*, *Gemella*, *Prevotella*, *Granulicatella*, *Porphyromonas*, and *Leptorichia*) identified using the DivCom clustering method.

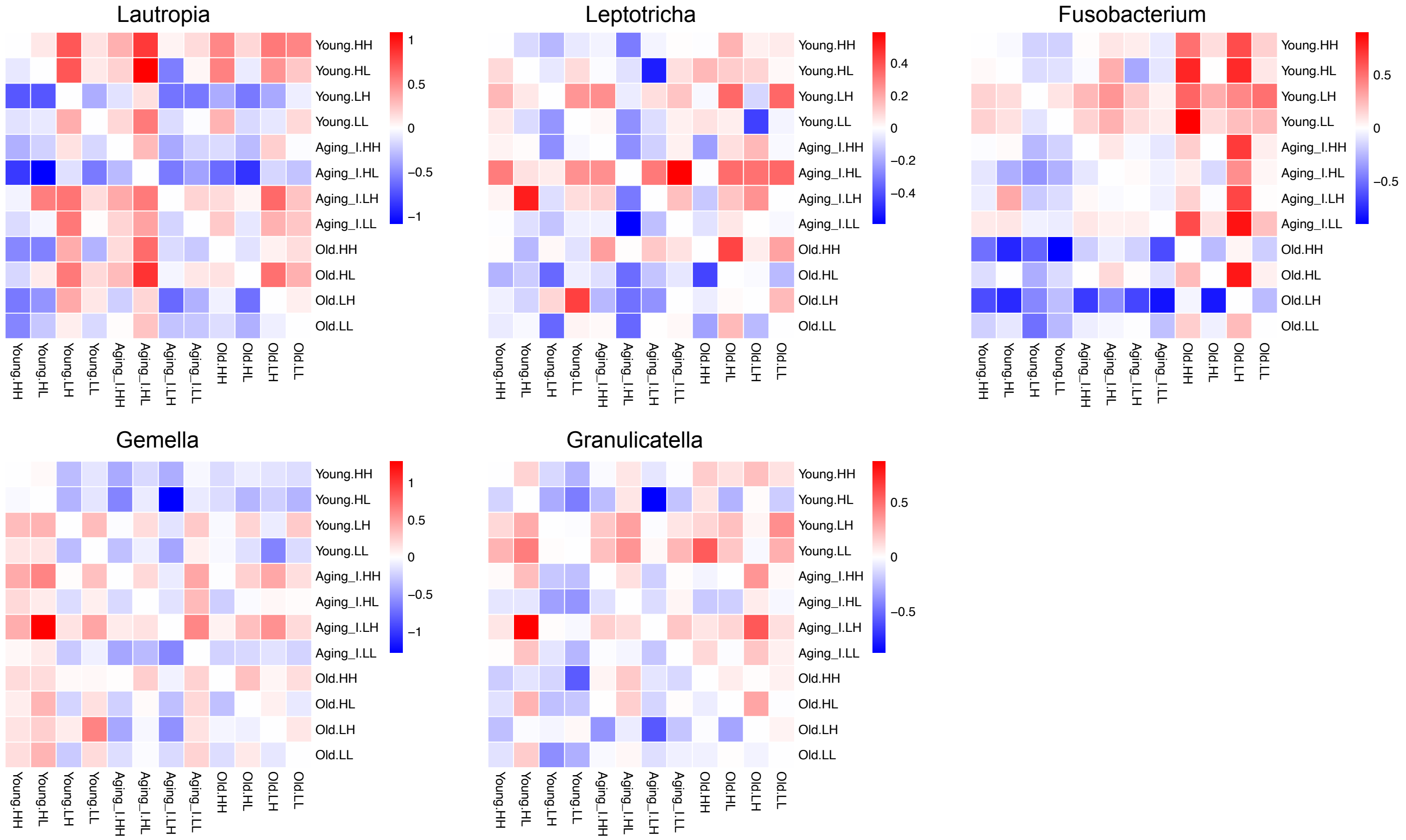

Supplementary Fig. 7. Heatmap of the pairwise comparison analysis for the five out of 16 core genera. The five of 15 core genera, the heatmap plots of negative log10-transformed p-values from all possible pairwise comparisons using ANCOM-BC2 were generated. Relative P-value differences between the two groups are marked with + (red) and - (blue). They all have different composition patterns, and the scale was set by their significance.
